# Biocompatible Macroion/Growth Factor Assemblies for Medical Applications

**DOI:** 10.3390/biom13040609

**Published:** 2023-03-28

**Authors:** Aneta Michna, Agata Pomorska, Ozlem Ozcan

**Affiliations:** 1 Jerzy Haber Institute of Catalysis and Surface Chemistry, Polish Academy of Sciences, Niezapominajek 8, PL-30239 Krakow, Poland; 2 Bundesanstalt für Materialforschung und-prüfung (BAM), Unter den Eichen 87, DE-12205 Berlin, Germany

**Keywords:** polyelectrolytes, polysaccharides, vascular endothelial growth factor, human fibroblast growth factors, neurotrophins, macroion/growth factor assemblies

## Abstract

Growth factors are a class of proteins that play a role in the proliferation (the increase in the number of cells resulting from cell division) and differentiation (when a cell undergoes changes in gene expression becoming a more specific type of cell) of cells. They can have both positive (accelerating the normal healing process) and negative effects (causing cancer) on disease progression and have potential applications in gene therapy and wound healing. However, their short half-life, low stability, and susceptibility to degradation by enzymes at body temperature make them easily degradable in vivo. To improve their effectiveness and stability, growth factors require carriers for delivery that protect them from heat, pH changes, and proteolysis. These carriers should also be able to deliver the growth factors to their intended destination. This review focuses on the current scientific literature concerning the physicochemical properties (such as biocompatibility, high affinity for binding growth factors, improved bioactivity and stability of the growth factors, protection from heat, pH changes or appropriate electric charge for growth factor attachment via electrostatic interactions) of macroions, growth factors, and macroion-growth factor assemblies, as well as their potential uses in medicine (e.g., diabetic wound healing, tissue regeneration, and cancer therapy). Specific attention is given to three types of growth factors: vascular endothelial growth factors, human fibroblast growth factors, and neurotrophins, as well as selected biocompatible synthetic macroions (obtained through standard polymerization techniques) and polysaccharides (natural macroions composed of repeating monomeric units of monosaccharides). Understanding the mechanisms by which growth factors bind to potential carriers could lead to more effective delivery methods for these proteins, which are of significant interest in the diagnosis and treatment of neurodegenerative and civilization diseases, as well as in the healing of chronic wounds.

## 1. Introduction

Macroions, also known as polyelectrolytes, are a type of charged polymer that contain charged groups bonded covalently to the polymer chain [1]. These macroions can be divided into two main categories: polyampholytes, which contain both anionic and cationic groups, and homogenous macroions, with only one type of charge [2]. In addition, macroions are classified as “weak” or “strong” based on their ionization constants. They can have a variety of shapes and polydispersity indices, and can be found naturally (e.g., in proteins and polysaccharides) or synthesized artificially. Macroions are water-soluble and can swell to bind large quantities of water molecules They are commonly used in industry as emulsifiers, thickeners, and flocculating agents, and can also serve as antifouling agents to prevent protein or bacterial adsorption or in the creation of effective antifouling coatings [3].

One of the main advantages of macroions compared to uncharged polymers is their ability to interact strongly with oppositely charged surfaces and macroions. The long-range electrostatic interactions between these charged species contribute to polyelectrolyte adsorption, which depends on various factors including adsorption energy, surface charge, molecule charge, as well as the ionic strength and the pH of the environment [4]. It is generally believed that macroion adsorption is an irreversible process and leads to a reversal of the substrate charge [5].

The irreversible process of adsorption, also known as deposition, is of significant importance in soft colloid science and various phenomena that occur in daily life. This ability to irreversibly adsorb onto surfaces makes macroions a useful tool in the layer-by-layer (LbL) method for creating thin coatings with controlled architectures on solid supports. In the LbL method, macroion multilayers (MM) are deposited by alternately adsorbing oppositely charged macromolecule chains [5]. A wide range of molecules, nanoparticles, and cells can be easily incorporated into the macroion layers, allowing for the synthesis of functional materials with desired compositions and architectures. Biocompatible macroion multilayers can be formed using both synthetic and natural polyelectrolytes. The formation of these multilayers depends on various factors including the type of macroion, solution type, solution ionic strength and pH, substrate characteristics, macroion layer rigidity and thickness, surface roughness, and post-assembly modifications. Over the past few decades, biomaterials formed from biocompatible MMs have been widely used in biotechnology for applications such as the encapsulation for controlled drug delivery and release [6,7], biosensing [8], and protein and enzyme immobilization and separation [9]. In medical applications, MMs were used as drug [10] and protein carriers [10,11], in particular as growth factors (GFs) [12,13] and inhibitors of bacterial growth [14]. In recent years there has been a rapid expansion in the use of polysaccharide-based MM due to their high biocompatibility and nontoxicity. These multilayers can be used as drug release vehicles or to generate surface-controlled cell adhesion. Most polysaccharides are negatively charged over a wide range of pH and ionic strengths, with chitosan being a notable exception as a positively charged polysaccharide. As a result, most polysaccharides readily interact with proteins such as positively charged growth factors [15].

It is worth noting that the formation of MM is closely related to the creation of macroion complexes, also known as complex coacervates (CC), in bulk [4]. CC are formed when two oppositely charged macroions, a macrocation and a macroanion, undergo associative phase separation in an aqueous solution [16]. This separation results in the formation of a macroion-dense coacervate phase and a macroion-dilute supernatant phase. Electrostatic interactions play a major role in the formation of CC [17]. The physicochemical properties of CC made of synthetic macroions have been extensively studied and reported to have potential applications in biotechnology and industry [18], in dialysis, ultrafiltration, seawater desalination, and other areas [19]. The use of natural macroions, particularly biocompatible polysaccharides, in the formation of CC has also received significant attention, with these assemblies being used in enzyme immobilization, protein purification [20], growth factor delivery [21,22], and food formulation [23]. The use of complex coacervates containing polysaccharides is also well-known as the oldest and most efficient method for encapsulating drugs and GFs [24,25].

The extracellular matrix (ECM) is a three-dimensional network of macromolecules such as glycoproteins, collagens, glycosaminoglycans (GAGs), and proteoglycans. The ECM provides structural and biochemical support to surrounding cells, serves as a substrate for cell migration, and plays complex and crucial roles in signaling events through various cell surface GF receptors and adhesion molecules such as integrins [26]. GAGs are functional determinants that encode a significant amount of information that cells can interpret to influence their metabolism and behavior, leading to the maintenance of tissue homeostasis and the modulation of their structure and the assembly of key ECM components in tissue morphogenesis [27]. Proteoglycans are complex, diverse extracellular or cell surface-bound macromolecules composed of a central core protein with covalently linked GAG chains [28]. Both the protein and GAG components of proteoglycans contribute to their many bioactive functions, including their role in nervous tissue development, which arises from dynamic interactions that modulate signalling fields for cytokines, growth factors, and morphogens that can bind to either the protein or GAG portions.

The interactions between ECM, GFs, and cells play a crucial role in tissue generation and regeneration [29]. GFs are signaling polypeptides that can stimulate specific cellular responses, including adhesion, migration, proliferation, differentiation, and gene expression [25,30]. Some proteins in the ECM have binding sites for both cell adhesion and cell surface receptors, allowing GFs to be localized near these sites. This GF localization, and the resulting signaling, helps to establish gradients of soluble diffusible GF morphogens [31,32], which are essential for the patterning of cell development processes. For example, fibroblast growth factors (FGFs) and vascular endothelial growth factors (VEGFs) bind to heparan sulphate proteoglycans (HSPGs) and can be cleaved off from the glycosaminoglycan components of HSPGs by the enzyme heparanase to be released as soluble ligands [33]. However, it has been discovered that most GFs cannot be directly applied due to their susceptibility to degradation in vivo, resulting in the loss of their biological activity [12,13,15]. Additionally, GFs have a short effective half-life and low stability, and they can be deactivated by enzymes at typical body temperatures (around 37 °C) [34]. Direct injection of a high dose of GFs to achieve and maintain a high local concentration may also have negative side effects in vivo, including an increased risk of cancer development [34].

This review focuses on recent literature discussing the physicochemical characteristics and applications of selected biocompatible macroions, GFs, and macroion-GF assemblies. These include synthetic macroions such as poly(diallyldimethylammonium chloride) (PDADMAC), poly(allylamine hydrochloride) (PAH) and its derivatives, poly(β-aminoesters) (PAEs), branched polyethyleneimine (bPEI), polyamidoamine dendrimers (PAMAM dendrimers), and poly(acrylic acid) (PAA), as well as natural macroions such as chitosan (CS), hyaluronic acid (HA), heparin, λ-carrageenan, and chondroitin sulfate (ChS). It is worth noting that ChS, HA, and heparin sulfate are GAGs that can stabilize and protect GFs in situ, increasing their biological half-lives [35,36]. Additionally, the properties of representative GFs including neurotrophins (NTs), FGFs, and VEGF are discussed.

The selected biocompatible synthetic macroions and natural polysaccharides have several well-established applications in medicine as carriers for the effective delivery and controlled release of growth factors. These biopolymers possess certain biological and physicochemical properties that are suited to their cargo, allowing for long-term effectiveness and controlled release. These properties are summarized in terms of typical bulk and surface conformations, isoelectric points (IEPs), zeta potentials, and typical sizes under different environmental conditions, the advantages and limitations of macroions for biomedical applications, the physicochemical and biological properties of biocompatible macroions, the potential applications of NTs in medicine, the potential applications of FGFs in medicine, and the applications of biomaterials with attached or incorporated GFs.

## 2. Macroions

Both natural macroions, particularly polysaccharides, and synthetic macroions can meet strict criteria for medical applications, including biocompatibility, biodegradability, non-toxicity, and non-reactogenic properties (i.e., an inability to produce a physiological response).

Synthetic macroions can be obtained through standard polymerization techniques such as addition, condensation, or ring-opening [37]. There have been recent efforts to develop new strategies that involve reactions carried out under mild and simple conditions and facilitate product separation, such as the “click” reaction [38], based on thiolene/thiolyn addition [39], copper(I)-mediated Huisgen’s 1,3-dipolar cycloaddition of azides and alkynes [40], and controlled free radical polymerization [41]. The latter synthesis type is also used to obtain graft macroions extending from the surface of a substrate [41], which can significantly improve surface biocompatibility and find applications in implant materials.

Natural macroions, including proteins and polysaccharides made up of repeating units linked by covalent bonds, can be found in a variety of organisms, including animals, bacteria, fungi, green plants, and algae [42]. Polysaccharides, which are produced by living organisms, can be directly extracted from biological and renewable sources. Despite their many advantages, these macroions also have limitations that should be considered. For the reader’s convenience, the main advantages and limitations of synthetic and natural (only polysaccharides) macroions are summarized in Table 1 and Table 2. Within these two groups they were arranged according to the surface charge (from positive to negative), the strength of the macroion (from strong to weak), and the chain shape (from linear, through branched, to spherical).

As can be seen in Table 1, the synthetic macroions possess exceptional physicochemical properties that allow their use as effective carriers of GFs. They are biocompatible, easily soluble in water, and are suitable solvents of various polarity, ionic strengths, and pHs. Furthermore, they have a narrow range of molecular mass in which they are very similar to proteins. On the other hand, the polysaccharides, as natural macroions, constitute perfect building blocks for forming the stimuli-responsive assemblies for in vivo applications. Furthermore, they are naturally degraded by different kinds of enzymes [56]. This feature is a great advantage in comparison to synthetic macroions.

Both types of the aforementioned macroions have some limitations, which are summarized in Table 1. The biggest disadvantages of synthetic macroions are harmful degradation products, high costs, and multistage synthesis. In the case of polysaccharides, the broad molecular mass range, high polydispersity index [4], and lack of solubility in organic solvents [52] are the major drawbacks. The macroions discussed in this paper are significant because they can form an efficient carrier that prevents protein and DNA degradation in vivo and safeguards protein activity [57,58,59,60,61].

Numerous pieces of evidence from both experiments and numerical simulations showed that like-charged macroions can attract each other via electrostatic forces [62]. Studies indicate the existence of like-charge attraction in strongly-charged systems, i.e., when multivalent counterions are present, macroions are highly charged, or the strength of electrostatic interactions is enhanced by maintaining the system at low temperatures or in a medium of a low dielectric constant [62]. On the microscopic level, the like-charge attraction can be realized through the following groups of mechanisms [63]: 

(I) A “counterions sharing” mechanism: When macroions are far from one another, the counterions are assembled into non-overlapping identical layers around them; their effect is to screen the macroions’ charge, leading to the weakening of the repulsive forces).

(II) A “charge fluctuation” mechanism: Fluctuations of charge on one macroion induce fluctuations of charges of the opposite sign on the other macroion. The interaction between these opposite charges leads to an effective attraction between the macroions.

(III) Depletion forces: The attraction results from the expulsion of counterions from the areas at the interface between macroions. This leads to an unbalanced ion concentration, which creates osmotic pressure. Depletion forces can also be caused by excluded volume effect, repulsion from the boundary between the media with low and high dielectric constants, or strong ion−ion correlations.

A proper selection of co-solutes and solution conditions plays a crucial role in the protein purification, drug delivery, food industry, and biotechnological applications, involving protein− polyelectrolyte complexation [64]. Such a biomacromolecular complexation occurs on the so-called “wrong side” of the protein isoionic point, where both the protein and the macroion are net like-charged. The recent work of M. Simončič et al. [64] provided mechanistic insights into the modulatory role of various salts and sugars in protein−macroion complexation under such conditions.

### 2.1. Biocompatible Synthetic Macroions

Many synthetic macroions are biocompatible, biodegradable and non-toxic. Weak hydrolyzable links, creating the backbones, are mainly responsible for macroion biodegradability [37]. These links can be broken down into monomer units acceptable to the human body. Thus, synthetic macroions are significant in various biomedical applications. Most of them are easily synthesized by chemical techniques in mild conditions. The final products are thoroughly cleaned [38,39,40], providing the possibility to receive macroions with a well-defined structure, molecular mass, charge, and mechanical properties.

However, the macroions used as GF carriers must meet additional criteria such as the facile preparation of macroion-GF assembly [13,21], the protection of cargo [21,22,25], improving cargo transport [45], maintaining bioactivity and protein stability [21,22,25], as well as sustaining their release [13,21,24]. PDADMAC, PAH and its derivatives, PAEs, bPEI, PAMAM dendrimers and PAA fulfill all of these criteria.

#### 2.1.1. PDADMAC

PDADMAC is often applied in biotechnology for dendronized polymer (DP) gelator formation [65] and water treatment [46]. It is deposited on a solid substrate serving as an “anchor layer” [66] used for producing multilayers (films) of various coverage and structure [67], in anion-exchange membranes for fuel cells [68], and in the design of dental materials [69].

PDADMAC is a strongly positively charged hydrophilic polycation because of the presence of the quaternary ammonium group. The positive value of the measured mobility unequivocally indicated that the electrokinetic charge of PDADMAC remained positive for the broad range of ionic strengths (0.0001–0.15 M). Adamczyk et al. also reported that the PDADMAC electrokinetic charge is considerably smaller than the nominal charge. The effective ionization degree varied between 13% and 8% for an ionic strength of 0.0005 and 0.15 M, respectively [70].

Other PDADAMC physicochemical bulk properties were described in the literature. The combination of molecular dynamics (MD), rotational isomeric states, and the Monte Carlo procedure revealed the chain conformations in a vacuum and pure water and various salt solutions. The trans conformation of the three rotatable skeletal CH–CH bonds of the chain units was favoured, leading to the formation of the extended macroion chains [71]. It was confirmed that the PDADMAC molecules remain expanded even for the high ionic strengths with a length-to-width ratio exceeding 36 [70]. For larger ionic strengths (*I* > 0.1 M), the random coil limit is attained [72]. The dependences of its intrinsic viscosity, the radius of gyration, and the second virial coefficient on ionic strength and the composition of electrolytes were also experimentally evaluated [70,72]. The experimental results confirmed the theoretical calculations [70].

For low ionic strength, PDADMAC molecules adsorbed in a “side-on” orientation and flattened substantially during adsorption. The obtained layers are thin. However, due to the strong attractive interactions acting between the macrocation chain and the substrate, the coiling of the PDADMAC prevails in high ionic strengths. The obtained layers are thicker and less dense [73]. Accordingly, the thickness and density of the PDADMAC layers can be easily tuned by changing the ionic strength of the PDADMAC solution. The major role of electrostatics in PDADMAC adsorption was also studied in Ref. [74]. Using the theoretical approach (correlation-corrected classical density functional theory for macroions) and the experimental method (ellipsometry measurements), the authors stated that the electrostatic interactions play a major role during the PDADMAC adsorption on a solid substrate. It is worth noting that the PDADMAC surface coverage increases progressively with ionic strengths up to 0.2 M [74].

#### 2.1.2. PAH and Its Derivatives

PAH is one of the best known and most frequently used macroions. PAH is a weak polybase with a high-pH-dependent charge forming a prolate spheroid in electrolytes of moderate ionic strength. In high ionic strengths it can be bent to a semicircle [75].

The literature data revealed that PAH molecules are irreversibly adsorbed on the solid substrate, and their chains are attached to a solid substrate in the “side-on” (flat) conformation [75,76]. The adsorbed PAH layer consisted of equal masses of PAH and water entrapped within this layer. Accordingly, the PAH monolayer usually consisted of an adsorbed dry mass of about 0.5−1 mg/m^2^, and a water content of 20–50%. The layer thickness increases with increasing salt concentration and pH. Therefore, the PAH layer can swell or shirk depending on experimental conditions (ionic strength, solution pH) [77]. The PAH layer was stable upon rinsing when the pH of the rinsing solution was the same as that used in the buildup [78]. However, the streaming potential measurements revealed that some PAH molecules desorbed from the mica substrate during extensive rinsing. It was also stated that the decrease in the zeta potential was less significant for the PAH layer than for the bPEI layer, indicating the higher stability of the PAH monolayer on mica [79].

Due to the pH-dependent properties, PAH is applied for the formation of biocompatible constructs in the efficient delivery of GFs [80]. The films containing a PAH layer are commonly used in bioimaging applications, drug and proteins delivery and release [18,81,82], as well as in supporting the adhesion of proteins and cells [76]. Some literature data suggest that PAH is toxic to cells, and using it as a carrier does not work effectively for safe and efficient gene transfection [83]. For improving transfection efficiency and to reduce the toxicity of native PAH, the PAH derivatives are synthesized by various chemical modifications [60,83]. Opposite to PAH, the PAH derivatives have been reported to work effectively as carriers for safe and efficient gene transfection [60] as well as drug delivery and release [84]. Moreover, the photoreactive EGF was synthesized by conjugating mouse EGF with a photoreactive PAH derivative [85].

#### 2.1.3. PAEs

**Figure 1:**
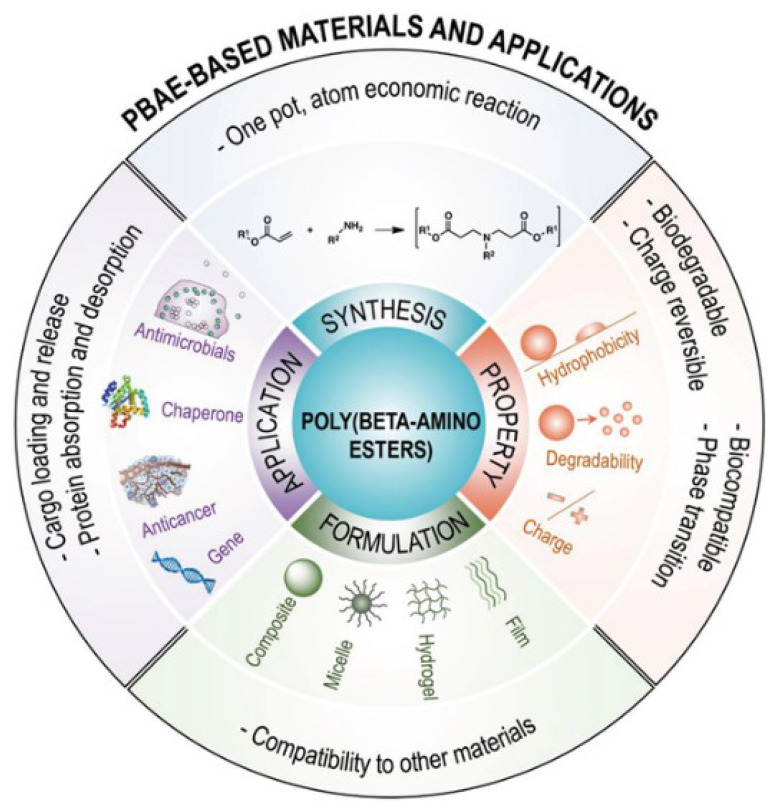
The properties of PAEs and PAE-based materials. Reproduced by permission of Copyright © 2018 WILEY-VCH Verlag GmbH & Co. KGaA, Weinheimfrom Ref. [86]. Poly(β-amino esters) (PBAE) are synthesized via a one-pot, atom-economic Michael addition of amines (NH_2_-R^2^) to acrylates (CH_2_=CHCOOR^1^) without the production of any side products.

D. M. Lynn and R. Langer [61] reported a preparation strategy of PAEs in 2000. They are mainly synthesized by a one-pot Michael addition of amines to acrylates without obtaining any side products [86] (Figure 1). Physicochemical properties of PAEs, such as molecular mass, polydispersity index (PDI), hydrophobicity and charge, rely heavily on the monomers employed in the polymerization [86,87]. Their molecular mass can vary from 2 to 120 kDa by tailoring the monomers and synthesis conditions [86]. PAEs obtained from the Michael addition polymerization usually have a relatively wide polydispersity (PDI > 1.3) [86] compared with other types of polymerizations, such as reversible addition-fragmentation chain transfer and atom transfer radical polymerization. PAEs possess tertiary amino groups; thus, they are highly positively charged within a wide pH range (from 3.5 to 7.2) [88]. Furthermore, the easy protonation of the amine groups makes PAEs hydrophilic [86]. PAEs are stable in acidic conditions; however, they easily degrade under basic/physiological conditions due to hydrolysis of the backbone esters [86]. PAEs also exhibit thermoresponsive and selective cell binding behaviour, as was shown by Zhou and co-workers [87].

Positively charged PAEs chains interact electrostatically with negatively charged therapeutic macroions, such as DNA [61,89] and peptides [90]. Thus, they can be applied as efficient degradable polymeric gene nanocarriers [91]. These polymers have been proven to be successful as potential biomaterials for tissue engineering scaffolds and depots for the sustained release of drugs [86].

PAE-FA containing folic acid (FA) and amino groups in the backbone and side chain was synthesized by P. F. Tsai et al. [92]. They found that all PAE-FA polymers were able to bind plasmid DNA. Those results further demonstrated that the introduction of FA into the poly PAEs system had a significant effect on the transferring ability of folate receptor (FR)-positive HeLa cells.

Finally, it should be mentioned that PAEs are biocompatible, biodegradable and noncytotoxic in opposition to common cationic polyelectrolytes (such as PLL) that can be significantly cytotoxic [93]. Hyperbranched poly(β-amino ester)s (HPAEs) have been developed as a class of safe and efficient gene delivery vectors [94,95,96].

The synthesis, main properties and applications of PAEs and PAE-based materials were presented in Figure 1.

#### 2.1.4. BPEI

BPEI is a weak polybase possessing primary, secondary and tertiary amino groups (in a 1:2:1 molar ratio) in contrast to linear polyethyleneimine (lPEI) containing only secondary groups. V. Kalif et al. have compared those two polymers in terms of cellular toxicity [97]. Based on this work, lPEI can be considered safer than bPEI, even though its transfection efficiency is lower than bPEI, as bPEI can induce greater cytotoxicity than lPEI. Despite the induction of the Akt-kinase pathway, bPEI treated cells exhibited DNA fragmentation.

The acid-base properties of bPEI were determined theoretically (using Isingmean field and site-binding models) [98] and experimentally by conductometric, potentiometric, and calorimetry titration [98,99]. Three protonation steps of bPEI were observed [98,99]. In the first step, occurring at pH 9.0–9.5, only the primary groups situated on the side chains are protonated; in the second step (at pH 4.5–5.0), all primary groups and every second tertiary amine group protonate; at the third step (pH range near 0), the remaining tertiary groups protonate. BPEI is not fully protonated under physiological conditions, even at pH 2. Its mean protonation degree significantly decreases with increasing pH [100].

BPEI has a spherical conformation in the solution; however, it is slightly flattened due to adsorption on a solid surface [101]. BPEI is polydisperse, which was confirmed in Ref. [102]. The presence of tertiary amine groups allows bPEI to act as the “proton sponge” [44], thus, it can be applied in biotechnology and medicine. For example, bPEI is used as a vector for plasmid DNA delivering to mammalian cells [103]. It provides greater protection with the cargo against enzymatic degradation compared to other polyamines [103], and serves as the GF carrier in cancer treatment [104]. Moreover, bPEI/antisense oligonucleotide (ASO) nanoconjugates (nanocarriers) were functionalized with a muscle-specific RNA aptamer [105]. Using this combinatorial formulation methodology, nanocomplexes were obtained for the delivery of RNA therapeutics, specifically into muscle cells.

#### 2.1.5. PAMAM Dendrimers

PAMAM dendrimers represent monodisperse, nano-sized, radially symmetric, charged macromolecules containing easily functionalizable surface groups. They have a well-defined structure possessing tree-like arms or branches [106]. The dendrimer structure is formed by three distinct parts: (1) a central core, (2) repeating branching units, allowing for macromolecule growth in organized layers and (3) the numerous terminal groups, which are created by diverse organic substituents.

Among each branching point, the PAMAM dendrimers form layers known as “generations”. The molecular size and the number of terminal surface groups increase with the generations. That allows the formation of various host-guest complexes with a broad range of applications. The physicochemical and biological properties of dendrimers can be improved by modifying the terminal functional groups (e.g., primary amines NH_2_^+^ or carboxylic groups COO^−^). Therefore, dendrimers seem to be an ideal delivery vehicle for the parametric study of the effects of macromolecule size, charge, and composition on biologically relevant properties such as lipid bilayer interactions, cytotoxicity, internalization, blood plasma retention time, biodistribution, and filtration [107].

Drugs, proteins, genes, and cells are protected from physiological conditions if they are entrapped within the dendrimer internal cavity or electrostatically combined with the dendrimer surface [108]. The PAMAM dendrimers enhance the permeation and retention effect and minimize the side effects of loaded drugs [109]. Thus, they are broadly exploited as the nanocarriers of genes [106,110] and drugs [106,109].

There are two main types of PAMAM dendrimers possessing primary amines NH_2_^+^ (PAMAM-NH_2_) or carboxylic groups COO^-^(PAMAM-COO) situated on the rim. If the-NH_2_^+^ are the terminal groups, the charge of PAMAM dendrimers can be easily tuned by pH changes. NH_2_^+^ groups, situated at the outer rim, protonate at high pH, the tertiary amine groups, forming PAMAM core, protonate at lower pH, and the central tertiary amine groups protonate at low pH [111]. The high number of tertiary amine groups gives the PAMAM-NH_2_strong pH buffering ability (pK~6.0). Therefore, the dendrimers act as a “proton sponge” [112]. The PAMAM-NH_2_ charge neutralization is significant over the broad pH range. For example, the generation 8 of PAMAM-NH_2_ possesses a low ionization degree, ranging from only 2.2 to 0.2% [50]. Using the small-angle neutron scattering (SANS) technique, Nisato et al. [113] and Porcar et al. [114] discovered that the PAMAM-NH_2_ gyration radius was independent of ionic strengths, charge density and pD. A minor dependence of the hydrodynamic diameter of the dendrimers on pH was confirmed by Michna et al. [50]. A slight change in the radius of gyration with pH was also predicted theoretically [115]. However, Welch and Muthukmar reported a significant increase in the dendrimer size for a lower ionic strength range [116]. Similarly, Lee and coworkers reported a significant increase in the dendrimer gyration radius for lower pHs [117].

Interesting results were obtained for the PAMAM-NH_2_ monolayers deposited on solid substrates. It was found that PAMAM-NH_2_ maximum coverage increases with pH and ionic strength [118,119]. The adsorbed dendrimers undergo conformational changes depending on ionic strength, pH and dendrimer generation [50,120,121]. The flattening of the dendrimers due to adsorption and the deformation degree depend on the pH of a solution, as was shown by Wolski and Panczyk [122], together with the formation of the more compact, compressed structure of the dendrimer layers with increasing pH.

The PAMAM-NH_2_ dendrimers are also very interesting due to their solvation phenomena leading to macroion swelling or shrinking. The dendrimer swelling, depending on pH, ionic strength, electrolyte type, generation or type of substrate, is still debated. The dendrimer solvation effects were studied both theoretically [115,123] and experimentally [118,119]. The existence of three types of bound water (buried water placed inside of the dendrimers, surface water situated on dendrimer-water interface and bulk water placed outside the dendrimers) were theoretically postulated by Maiti et al. [115] and Lin et al. [123]. According to Maiti et al. [115], the water content increased with the generation and decreased with pH. The water entrapped within the PAMAM-NH_2_ dendrimer monolayer was also studied using the quartz crystal microbalance (QCM-D) technique [118,119,120,121]. It was found that the total hydration exhibits a tendency to increase for lower pHs, and it amounted to 80% and 70% for gold [118] and silica [119], respectively. Mureşan et al. [120] and Porus et.al. [121] stated that the hydration of the PAMAM-NH_2_ dendrimer covered silica increased with ionic strength. The hydration of the tenth generation PAMAM dendrimer monolayers on silica was found to be in the range of 50–80% [121].

PAMAM-NH_2_ dendrimers are widely used in the biomedical sciences. Haensler and Szoka proved that the PAMAM-NH_2_ dendrimers effectively induce the transfection of genes (luciferase and beta-galactosidase containing plasmids) in both dispersed and deposited cultured mammalian cells, whereas PLL caused cell death [124]. Various generations of PAMAM-NH_2_ were also applied for the in vitro transfection of mesenchymal stem cells. The transfection efficiency was very low and dependent on the generation of dendrimers, the amine-to-phosphate group ratio, and the cell passage number. However, the low transfection efficiency was found to be sufficient for inducing the in vitro differentiation of mesenchymal stem cells to the osteoblast phenotype [125]. PAMAM-NH_2_ dendrimers were also applied as effective carriers of anticancer drugs [126] and in the treatment of lung diseases [110]. It was also demonstrated that PAMAM-NH_2_ dendrimers possess the ability to disturb the process of amyloidogenesis; thus they can be effectively applied in the treatment of Alzheimer’s disease [127]. PAMAM-NH_2_ dendrimers were also used in the treatment of skin wounds [128] or infections [129].

However, it is reported in the literature that some PAMAM-NH_2_ dendrimers exhibit charged–related cytotoxicity [130], causing platelet aggression [131], and such dendrimers are removed from the body by the clearance by the reticuloendothelial system (RES), which limits their use as carriers [130]. It should be mentioned, that the dendrimer cytotoxicity depends on the concentration, dendrimer charge and generation. The lower generation PAMAM-NH_2_ dendrimers are reported to be less toxic than the higher ones [132,133]. High-generation PAMAM-NH_2_ dendrimers had an impact on the mitochondrial activity, apoptosis, and neuronal differentiation of human neural progenitor cells (hNPCs). In particular, the high surface charge of these dendrimers adversely affects the cell viability and neuronal differentiation of hNPCs [133].

When PAMAM-NH_2_ dendrimers are modified with polyethylene glycol (PEG) or acetyl groups, they lose the positive charges on their surface and their cytotoxicity is significantly reduced. However, the charge reduction is also responsible for decreasing the transfection efficacy of the DNA [134]. The transfection efficacy can be increased by dendrimer surface modifications. For example, combining guanidinobenzoic acid molecules to PAMAM-NH_2_ dendrimers leads to the formation of modified dendrimers of high efficacy in both siRNA and DNA delivery, while the phenyl groups could induce efficient endosomal escape [135].

Half-generation PAMAM dendrimers possessing carboxylate groups situated on the rim were reported to be much less cytotoxic than most PAMAM-NH_2_ [133]. PAMAM-COO dendrimers were not haemolytic towards a panel of cell lines in vitro, as was shown by Malik et al. [132]. They did not induce platelet aggregation and did not change the function of platelets or their morphology, irrespective of their generation [131]. The PAMAM-COO dendrimers have been successfully used as an anticancer drug carrier [109]. They inhibited antimicrobial activity in intraamniotic infection [136] and significantly increased the solubility of poorly soluble drugs [137].

The physicochemical properties of the PAMAM-COO have not been systematically studied so far. It was reported that both the hydrodynamic radius of PAMAM-COO [131], in addition to the zeta potential [131], determined for the ionic strength of 0.01 M NaCl and undefined pH, and increased with the dendrimer generation. The surface charge of generation 3.5 PAMAM-COO was evaluated by the small-angle X-ray scattering method (SAXS) [138,139]. Micali et al. found that the PAMAM-COO is only partially ionized in methanol solution with an effective charge equal to 6, independently of the macroanion bulk concentration [138]. In water, the bulk properties of the same generation PAMAM-COO were also determined by Lombardo et al. [139] and Vu et al. [109]. The radius of gyration was constant (equal to 2.01 nm) independently of the dendrimer concentration in the bulk, whereas the effective number of ionizable groups, albeit slightly dependent on the dendrimer concentration and the ionization degree of carboxylic groups, was near 40% [139]. These dendrimers were negatively charged in water (the zeta potential was equal to −50 mV), and the diameter, obtained from the transmission electron microscopy, was equal to 5.68 nm [109]. PAMAM-COO layers were flattened and compressed within the multilayers [140] and formed homogenous layers on amino silylated glass slides [141]. The surfaces coated with PAMAM-COO were more hydrophilic than those covered by PAMAM-NH_2_ [142]. The hydrophilicity of the PAMAM-COO layer in protein microarray increased with the dendrimer generations (for generations 1.5−4.5) [141]. The PAMAM-COO monolayer charge depended on pH. It decreased until pH 5, then increased to 8.3, and then decreased again to a negative value of zeta potential [142]. It has been reported by Schilrreff et al. that PAMAM-COO caused a selective and concentration-dependent cytotoxicity to melanocytes (SK-Mel-28 cells) [143].

#### 2.1.6. PAA

PAA is a weak, linear polyacid possessing a middle ionization constant pKa (α = 0.5) of about 5.5 [144]. The structure of the PAA in bulk was studied experimentally [145] and theoretically [145,146]. The MD calculations revealed that the shape of PAA chains strongly depends on the ionization degree, ionic strength and pH of the solution. For fully nominal ionization and low electrolyte concentration, the macroanions assume the shapes of flexible rods with effective lengths comparable with the contour lengths of fully extended chains. For lower ionization degrees and high ionic strength, the PAA molecules assume the shapes of spheres [145].

The dependence of the nominal ionization degree of PAA on the macroion shape was confirmed by Batys et al. by MD dynamics and a modified mean-field Poisson-Boltzmann model [146]. It should be noted that the effective lengths derived from experiments (viscosity measurements) were in good agreement with values predicted from the MD simulations [145]. The detailed physicochemical properties such as hydrodynamic radius, radius of gyration, molecule volume, and the extended length of PAA of various molecular masses were investigated by Adamczyk et al. [147].

The cytotoxicity and intracellular effects of PAA were evaluated with L1210 progenitor leukaemia cells and L6 myoblast cells [148]. The authors found that the macroanions interact with serum proteins and reveal a dose-dependent cytotoxicity on the leukaemia cells.

The mechanism of PAA adsorption on solid support was also determined. Accordingly, the adsorption PAA of higher molecular mass was three-dimensional with the contribution of loops and tails, whereas low molecular mass PAA adsorbed rather in a flat, “side-on” conformation [147]. The adsorption process of PAA on various substrates such as modified mica [147], silica [149], TiO_2_ nanoparticles [150] and ZrO_2_ particles [151], leading to the formation of the PAA monolayers, was also studied. The obtained results showed that adsorbed PAA monolayer is stable during rinsing [147], and the thickness of the layer increases with the ionic strength and polyelectrolyte molecular mass [150,151].

Due to the pH-tunable charge density of PAA, the multilayers based on this macroion show exponential growth [152]. High molecular mass branched (HMW) bPEI and PAA were utilized as a model for weak polyelectrolytes to investigate the growth mechanism and the drug loading/release of the multilayers by Yuan et al. [152]. It was discovered that the fabricated films possess a pH-triggered switchable polarity and tunable charge density associated with the outermost layer which can facilitate the loading of anionic or cationic drugs, offering a broad range of pH-controlled release rates and ultra-long release times. The application of PAA-based multilayers in medicine was also studied by Psarra et al. [153]. The authors used PAA brushes biofunctionalized with GFs to create an active cell culture substrate. It was stated that the covalent immobilization of the GF molecules onto the brush substrate enhances the biological response, even with lesser GF amounts than those contained in soluble culture media. PAA brushes with thickness of 30 or 15 nm also facilitate mast cell adhesion [154]. It was found that the specific ligands for cell-surface receptors can be covalently attached to the brushes. This provides a spatially controlled means of activating cells. Accordingly, the mast cell signaling can be investigated with patterned features of PAA conjugated with 2,4-dinitrophenyl groups that specifically bind and tors anti-2,4-dinitrophenyl IgE bound to high-affinity cell-surface receptors FcεRI.

PAA-based films swell easily. The swelling degree increases with pH and ionic strength [149]. PAA-based hydrogels are applied in medicine, for example for growth factor storage, delivery and release [155]. Furthermore, PAA-based hydrogels also possess mucoadhesive properties, and thus they are also used as artificial tears to treat dry eye syndrome [156].

### 2.2. Polysaccharides as Examples of Biocompatible, Natural Macroions

Polysaccharides belong to a group of macroions composed of repeating monomeric units of monosaccharides covalently linked to each other through glucosidic linkage [157]. Due to the ubiquitous occurrence of polysaccharides in nature [45], their high biodegradability combined with biocompatibility [45], and their feasible functionalization through a variety of chemical and enzymatic methods [157], they are ideal candidates for drug delivery and release [157], inhibitors of viruses [158] or as thickeners in food production [159].

Among a wild range of applications, the main advantage of polysaccharides is the possibility to apply them as the carriers of GFs [21,24,25,59]. CS, heparin, HA, λ-carrageenan and ChS seem to be the most promising candidates for the formation of macroion/GF assemblies for medical applications. Therefore, they are briefly introduced in the following sections.

#### 2.2.1. CS

Chitin is a natural macroion and one of the most abundant polysaccharides in nature that exists in the shells of crabs, shrimps, insects, algae and bacterial cell walls [160]. Thus, this easily accessible and low-cost biopolymer has high potential as a biological material. Chitin, composed of repeating *N*-acetyl-*D*-glucosamine units linked by *β*(1 → 4) glycosidic bonds, is insoluble in an aqueous solution [161]. It has a degree of acetylation of 100% [161]. The degree of acetylation (DA) is defined as the proportion of *N*-acetyl-*D*-glucosamine units to the total number of units [162]. Chitin is transformed into CS to increase solubility through enzymatic or chemical deacetylation [163]. If the polysaccharide possesses a degree of deacetylation larger than 50%, it is referred to as CS [164].

CS consists of randomly distributed *N*-acetyl-*D*-glucosamine and *D*-glucosamine units linked by *β*(1 → 4) glycosidic bonds [161,165]. One of the main advantages of CS is its easy solubility in an acidic medium due to the presence of amino groups, which can be protonated [165]. CS is poorly charged and insoluble at high pH. The literature data show that pH values separated by the soluble and insoluble states of CS lie between 6.0 and 6.5. The region of CS solubility depends on the ionic strength of the solvent and the polysaccharide DA [161]. CS pKa ranges from 6.0 to 7.3 [166,167].

CS conformations in aqueous solutions were studied both theoretically (by MD and coarse-grained (CG) models) [164] and experimentally by means of viscosimetry and size exclusion chromatography coupled to multi-angle laser light scattering (MALS) [168], allowing for the determination of the radius of gyration and the persistence length for different ionic strengths, pHs, chain lengths, and degree of deacetylations [164,168]. The conformation of CS depends on molecular mass, DA, ionic strength, pH, and temperature. For example, the high DA of CS leads to an increase in the rigidity of the polymer chain, whereas an increase in the ionic strength and pH of the solution leads to a more flexible conformation. Depending on the environmental conditions, CS can form rods, random coils and stiff coils [168]. Positively charged CS adsorbs on negatively charged surfaces such as mica [169], modified gold [53], silica [170] and emulsion droplets [171]. It irreversibly adsorbs in flat conformation, forming rigid and thin monolayers at low pH. At neutral pH, the strong swelling of the adsorbed CS layer is observed, and the layer becomes significantly thicker and forms gels [169,170]. The CS layers are hydrophobic with water contact angle values around 110°; however, plasma modification reduces the water contact angles to 56° [172].

CS has unique biochemical properties such as biocompatibility, biodegradability, non-toxicity, and it acts as a biological adhesive, as well as having antimicrobial and biological qualities [169,170]. Hence, CS and its derivatives have found a wide spectrum of applications in medicine, cosmetics, wound dressings, biochemical separation systems, tissue engineering, cancer diagnosis, etc. [163] Moreover, hydrophilicity and a net cationic charge enable CS to be a suitable polycation for the delivery of active ingredients such as drugs, growth factors, stem cells and peptides [160]. CS enhances surface-induced thrombosis and blood coagulation, as well as improving coagulation in vivo by influencing the activation of platelets. This polysaccharide is a hemostat, which means that it helps in natural blood clotting and blocks nerve endings and therefore reduces pain [173]. It is also worthy of note that CS-based hydrogels and nanoparticles play a major role in biomedical applications. The hydrogels activate macrophages for tumoricidal activity [174] and are applied in various stages of wound healing [160] and regenerative medicine [175]. On the other hand, CS-based nanoparticles were tested as safe carriers of drugs to treat ovarian cancer [176].

#### 2.2.2. HA/Hyaluronan

HA is a linear, hydrophilic glycosaminoglycan composed of *N*-acetyl-*D*-glucosamine and *D*-glucuronic acid units combined with regularly alternating *β*(1 → 3) and *β*(1 → 4) glycosidic bonds. Both units are in the *β* configuration, which allows all of HA bulky functional groups such as hydroxyls, carboxyl, acetamido and anomeric carbon to be in a sterically favourable equatorial position, whereas all of the small hydrogen atoms are in the less sterically favourable axial positions. Accordingly, the structure of the disaccharide is energetically very stable [177]. The free rotation around the glycosidic bonds of the HA backbone is limited, resulting in a rigid conformation of the molecule [178].

HA conformation in solutions depends on the local environment, including pH, ionic strength, specific ion interactions, local dielectric constant, excluded volume effects, or the presence of interacting moieties (e.g., proteins) [179]. However, it is believed that HA exists in solutions as crowded random coils [179] that trap approximately 100 times their weight in water [177]. At solution pH 2.5 in the presence of salt, HA molecules show the capability for self-association in the formation of a viscoelastic putty state, whereas at pH 2.5 in a mixed organic/aqueous solution containing salt they form gels [179].

The equilibrium between HA synthesis and degradation plays a crucial role in the regulatory function of the human body [177,178,179]. It determines its amount as well as hyaluronan molecular mass. Thus, two types of HA-HMW and low molecular weight (LMW) can be established. HMW HA (≥10^6^ Da) is anti-angiogenic, as it can inhibit endothelial cell growth [178]. It acts as a lubricating agent in the synovial joint fluid due to its viscoelasticity to mainly protect the articular cartilage. HMW HA can be cleaved into LMW HA (2 × 10^4^–10^6^ Da), which has been shown to possess pro-inflammatory and pro-angiogenic activities. This degradation of HMW HA into LMW HA occurs during some environmental and pathological conditions, such as asthma, pulmonary fibrosis and hypertension, chronic obstructive pulmonary disease, and rheumatoid arthritis [178].

The adsorption of HA chains on surfaces is mainly governed by electrostatic interactions and hydrogen bond formation. Furthermore, it strongly depends on solution pH, ionic strength, and type of substrate [180,181]. Ordered and associated structures have also been observed for HA on the surfaces, as reported by Cowman et al. [179]. No covalent bonds exist between HA and proteins as well as cells, which is uncommon among other GAGs present in the human body, such as ChS and heparin [182]. Accordingly, HA does not form the glycoconjugates, i.e., proteoglycans. HA can improve the biocompatibility of coated materials by enhancing the adhesion of certain types of cells with specific receptors to the coated material [181].

This macroanion has been examined for the delivery of drugs; dermal, nasal, pulmonary, parenteral, liposome-modified, implantable devices and genes; and applied in anticancer therapy [183,184]. Moreover, HA is a promising candidate to treat osteoarthritis because it spontaneously forms biocompatible nanoparticles which are able to control inflammation with a long-lasting action [178]. The HA-based hydrogel exhibited good antibacterial properties to effectively prevent wound infection due to the addition of an antibiotic [185].

#### 2.2.3. Heparin

Heparin is a highly negatively charged GAG of high polydispersity and proven biological activity [186]. It is involved in cell adhesion, migration, proliferation and differentiation, well-known as an effective anticoagulant and anti-inflammatory agent [186,187,188]. It is applied in lipid transport, clearance, wound healing [186], and is used for binding the FGFs and VEGF [186,188]. Heparin inhibits angiogenesis, which is critical for cancer progression [189]. Depending on protein charge, heparin can increase cell adhesion and protein adsorption [76] or inhibit protein adsorption [190]. The inhibition of protein adsorption has an important impact on blood protein prevention and can be applied in the development of vascular medical devices [190]. The main advantage of heparin is its ability to covalently attach to native proteins, which leads to proteoglycan formation. Accordingly, the availability of a GF for its receptor can be modulated not only by the ECM but also on the cell surface through binding to heparin sulfate proteoglycans, such as syndecans [191].

**Figure 2:**
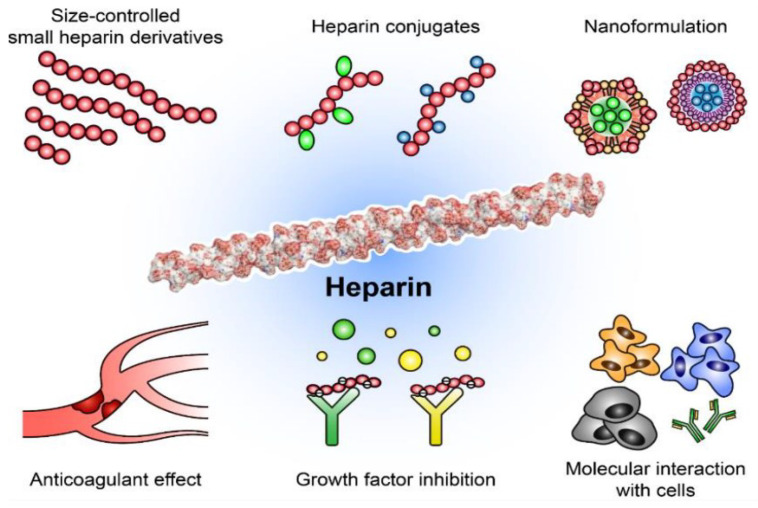
Schematic illustration of heparin and its derivatives applications. Reproduced with permission of MDPI from [188].

The application of heparin and its derivatives are summarized in Figure 2.

Heparin molecules consist of repeating uronic acid (*β*-*D*-glucuronic or *α*-*L*-iduronic) and *D*-glucosamine subunits linked by 1 → 4 bonds [187,192]. Uronic acid residue can be unsubstituted or sulphonated at the 2-*O* position, whereas the glucosamine residue may be either unsubstituted, sulphonated or acetylated at the amino group [192]. As a result, the heparin monomeric unit can be either nonsulphonated or contain anywhere from one to even three sulphate groups.

Heparin is biosynthesized using various enzymes by basophils and the mast cells of connective tissues [192]. The molecular mass of natural heparin is in the range of 5–40 kDa; however, the fraction of the range of 12–15 kDa is the most common [186,192]. Low molecular mass heparin (LMWH) is synthesized either by chemical or enzymatic depolymerization of commercial-grade heparin [193]. It allows for overcoming the poor predictability of coagulation parameters of natural heparin. LMWH can be intravenous or subcutaneously administered, which improves its therapeutic applications. LMWH is successfully applied to protect the FGFs from inactivation by heat and proteolysis. Furthermore, LMWH prolongs FGFs biological half-life and biological activity [57]. The molecular mass of LMWH is in the range of 4–6.5 kDa [57,192,193]. The tendency of heparin to produce complexes with magnesium and calcium was investigated by Yamane et al. [194]. It was shown that the binding ability of calcium ions to heparin was more efficient than that of magnesium, and the coexistence of these two metals reduced the binding affinity of each metal. The authors stated that this heparin ability can have an impact on its anticoagulant properties.

Due to the high charge density on the heparin chain (−75 for the molecular mass of 15 kDa), heparin is an elongated polyanion [186,195] without a tendency to coil. The hydrodynamic parameters such as the sedimentation coefficient, the translational diffusion coefficient, the intrinsic viscosity, the Kuhn segment length, and the hydrodynamic diameter of various molecular masses of heparins were determined for the high ionic strength of 0.2 M NaCl [195].

Heparin is applied for the efficient modification of positively charged substrates [76,190]. Multilayers containing heparin show exponential growth [190]. The incorporation of heparin increases the hydration level of the macroion-based films, making them softer (more dissipative) [190].

#### 2.2.4. λ-Carrageenan

Carrageenans are non-toxic, biocompatible polysaccharides of high viscosity and good solubility in water. They are broadly applied in drug delivery and release [196] as inhibitors of viruses [158,197] (even against severe acute respiratory syndrome coronavirus 2 (SARS-CoV-2) [198]) and bacterial infections [199]. The linear macroions possess backbones formed by *α*(1 → 3) and *β*(1 → 4)-linked galactose residues with repeating sulphate half-ester groups and 3,6-anhydro-bridges [200].

They are mainly obtained from red seaweed extract, which is a mixture of various forms of carrageenans [201]. The composition of the mixture depends on the algal source, life stage and even extraction procedure [202]. λ (lambda), κ (kappa), ι (iota), μ (mu), θ (theta) and ν (nu) are the main forms of carrageenans differing in the position and the number of sulphate groups within the disaccharide repeat units and in the content of 3,6-anhydrogalactose residues.

λ-carrageenan is especially interesting in terms of its structure and possible applications. It is mainly isolated from red seaweed *Gigartina skottsbergi* and *Sarcothalia crispate* [203]. It practically has no anhydro-oxygen bridge residues; therefore, it does not form a helix structure. λ-carrageenan does not gel because of the lack of 3,6-anhydrogalactose residues [159,200,204]. Analytical ultracentrifugation, light scattering, size-exclusion chromatography, and capillary viscometry revealed that it possesses a large molar mass in the range of 300–1000 kDa [204,205].

**Figure 3:**
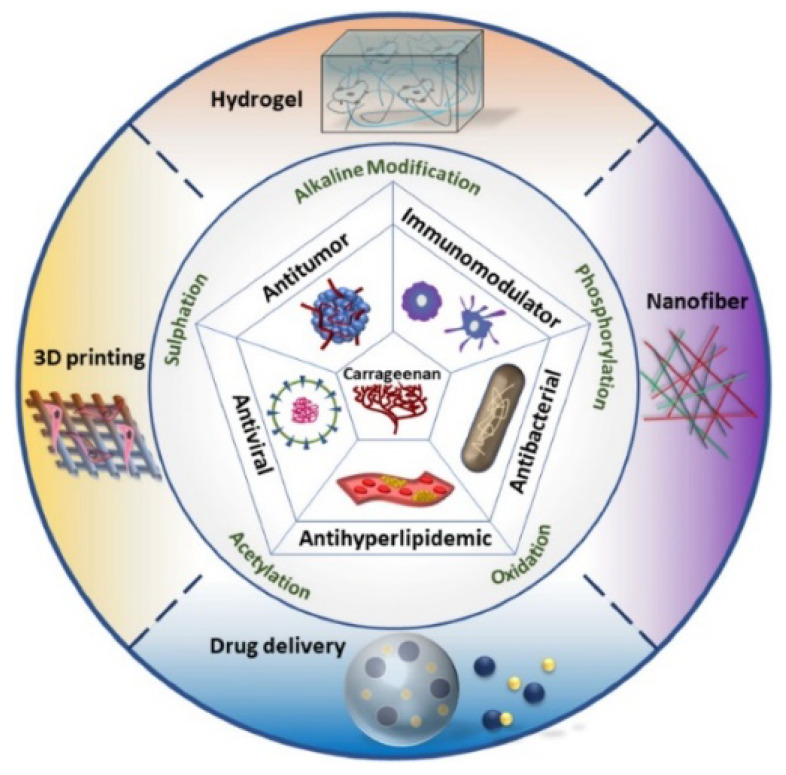
Applications of carrageenans in tissue engineering and regenerative medicine. Reproduced from Ref. [209], Copyright © 2021 Elsevier Ltd. All rights reserved with permission of Elsevier.

λ-carrageenan contains three sulfate groups per disaccharide unit and has a larger negative charge compared to other carrageenans. It is the most soluble type of carrageenan [204], producing viscous solutions exhibiting shear-thinning and pseudo-plasticity during stirring or pumping [200]. The aforementioned properties lead to its high solubility, even in cold water. λ-carrageenan is mainly used as a thickener for the stabilization of food products [159]. Furthermore, due to its antitumor and immunomodulation activities [206], it is applied in drug delivery and release [196], as an efficient agent preventing human papillomavirus (HPV) infections [158] and inhibiting the human immunodeficiency virus (HIV) [197], and promoting apatite formation [207]. It should be also underlined that λ-carrageenan is reported to protect growth factors against denaturation [59], and to antagonize the binding of some GFs [208]. The examples of carrageenan applications in medicine are summarized in Figure 3.

Because of its significance, the solution properties have been studied to evaluate its molar mass distribution [204,210], the radius of gyration, the contour length [211], persistence length [205], the hydrodynamic radius, and the second virial coefficient [210]. Several works have also focused on the determination of the intrinsic viscosity of solutions in various electrolytes comprising multivalent ions [212,213]. Interesting results were obtained by Berth et al., who analysed the polysaccharide solutions by MALS in 0.1 M NaNO_3_. It allowed the determination of the molar mass of the λ-carrageenan (1400 kDa), the radius of gyration (102 nm), and the second virial coefficient (10^−4^ mol ml g^−2^) [205]. The obtained data were interpreted in terms of the wormlike chain model using the Skolnik-Odijk-Fixman approach. Both the intrinsic persistence length of 2.7 nm and the expansion factor of 1.6 were also calculated. Physicochemical characteristics involving the molar mass, intrinsic viscosity and sedimentation coefficient (at pH 7.0 and ionic strength of 0.1 M) were reported by Almutairi et al. using size exclusion chromatography coupled to MALS, capillary viscometry, and analytical ultracentrifugation [204]. An extended and flexible conformation for the molecules was confirmed by these investigations.

Polyelectrolyte multilayers based on λ-carrageenan also show antibacterial properties, as was reported by Briones et al. [199]. The obtained films, analyzed by means of atomic force microscopy (AFM), X-ray photoelectron spectroscopy (XPS), and biomolecular interaction analysis, were effective in inhibiting the growth of *Enterobacter cloacae*. λ-carrageenan based multilayers were produced on various solid substrates such as mica [199], gold [214,215], silica [216], clay [217] or nanoparticles [218]. The films were studied by means of AFM [199,214,216], XPS [199,214], ellipsometry [216,217], polarimetry [216], circular dichroism [216], transmission electron microscopy [217,218], electrophoresis [218], contact angle measurements [214] and QCM [215,217] techniques. These studies allowed the determination of the structure of the coatings [216], the zeta potentials of nanocapsules based on the polysaccharide [218], the topography and roughness of the multilayers [214], or the oxygen permeability of the multilayers [217].

It was found that λ-carrageenan can form both loosely and highly packed structures depending on the anchoring layer type [219]. For example, the adsorption of λ-carrageenan on the PAMAM dendrimer layer leads to a heavier and more viscous/soft bilayer than the one built on the bPEI layer. Moreover, the λ-carrageenan chains tend to adsorb in the “side-on” conformation for low initial bulk concentrations of the polysaccharide, whereas for high bulk concentrations, the “end-on” conformation is preferred, and the adsorbed polysaccharide chains tend to form highly hydrated quasi “polymeric brushes” [219].

#### 2.2.5. ChS

ChS naturally occurs in the extracellular matrix of connective tissues such as bone, cartilage, skin, ligaments and tendons. It is an anionic, linear polysaccharide that is structurally similar to heparin. ChS comprises repeating disaccharide units of *D*-glucuronic acid and *N*-acetyl *D*-galactosamine linked by *β*(1 → 3) glycosidic linkages and is sulphated in various carbon positions [220]. Depending on the position of the sulfate group, ChS is divided into five main subgroups: ChS-A (chondroitin-4-sulfate), ChS-B (chondroitin-2,4 sulfate/dermatan sulfate), ChS-C (chondroitin-6-sulfate), ChS-D (chondroitin-2,6-sulfate) and ChS-E (chondroitin-4,6-sulfate) [221]. The zeta potentials of the various subgroups are negative (−17 to −40 mV) and practically independent of the sulfation degree of ChS [222]. Its molecular mass is also highly variable due to the different numbers of the disaccharide unit forming each ChS chain. Usually, the molecular mass of the naturally occurring ChS attains values between 50–100 kDa [223]. The extraction process used for obtaining commercial ChS results in some degradation of the molecular mass. Thus, commercially available ChS has a lower molecular mass in the range of 10–40 kDa.

Due to its negative charge, ChS is applied for biocompatible multilayer formation. ChS-based bioactive multilayers are homogenous, crack-free and well attached to the substrate even after bending [224]. Furthermore, ChS-based films reveal viscoelastic character and a tendency to form a three-dimensional scaffold. Furthermore, the multilayers can be applied as a potential trap for Ca^2+^ and PO_4_^3−^ ions inducing calcium phosphate precipitation, which is important in bone tissue engineering to improve implant osseointegration [225]. ChS is biocompatible and bioactive. It possesses anti-inflammatory, antithrombotic, antioxidant, anticoagulation and immunomodulatory properties. ChS enables the hydrating of the tissues. Therefore, it is applied as a nutritional supplement and drug for osteoarthritis treatment, tissue engineering and wound healing. It is believed that it can be successfully used in the treatment of cancer, cardiovascular diseases, as well as joint related pathologies [221]. As was stated in Ref. [222], the sulfate distribution within the disaccharide repeating units plays an important role in the binding of positively charged GFs [34,222,226].

#### 2.2.6. Protein-Polypeptide Nanoparticles

Polypeptide/protein nanoparticles have emerged as powerful tools for biomedical applications. They are biodegradable, biocompatible, cost-effective, easily metabolizable, and can be modified with cell-specific ligands, drug molecules, and GFs [227,228]. The ligands are bound to the protein nanoparticles by covalent or non-covalent bonds. Because of their non-antigenic properties, the nanoparticles can be used in cancer therapy [229] and in drug/vaccine delivery [230].

Due to their small size, polypeptide/protein nanoparticles can pass in the cells via endocytosis [231]. Apart from their small size (less than 200 nm, spherical shape), they have to be highly charged to prevent particle aggregation, safe to use in vivo, possess an acceptable shelf life, and reveal slow degradation to prevent sudden drug release. Furthermore, the degradation products should be easily metabolized and cleared from the body [228].

In GF delivery, the nanoparticles generated using the proteins, such as gelatin, fibroin, albumin, gliadin, and ferritin are particularly important. These proteins are extracted from a variety of natural sources such as recombinant protein expression systems, animals, plants and insects [228]. Electrospraying, emulsion/solvent extraction, salt precipitation, and macroion complexation are commonly applied for their preparation [228,231]. Gelatin nanoparticles were successfully applied as carriers of FGF2 and bone morphogenetic protein-2 (BMP-2), where the release of both therapeutics was observed, resulting in an inhibitory effect on osteogenesis [232]. Silk fibroin nanoparticles revealed the potential application as VEGF carriers. When VEGF was loaded on silk fibroin nanoparticles, a significantly sustained release of this GF over 3 weeks was observed [233]. The bovine serum albumin nanoparticles modified by PEI were tested as efficient carriers for the delivery of BMP-2 for in vivo bone induction [234]. The authors found that the PEI concentration used for nanoparticle synthesis efficiently controlled the release of BMP-2. Human ferritin-based nanoparticles were successfully conjugated with EGF. The obtained complexes possess narrow size distribution and small sizes of 11.8 nm. The authors found that they specifically bind to and are then taken up by breast cancer MCF-7 cells and MDA-MB-231 cells, but not normal breast epithelial MCF-10A cells. These nanoconstructs are very promising for clinical applications due to their reasonable biosafety and in vivo tumour accumulation [235].

The physicochemical and biological properties of the biocompatible macroions are summarized in Table 3.

### 2.3. GFs

GFs are polypeptides or proteins which control cell growth, differentiation, metabolism, and regulate the process of tissue repair [25,30]. They also modulate cell migration, adhesion, and gene expression [239]. GFs can have either positive or negative influences on these processes, and their activities vary with cell type and the developmental stage of the organism. ECM can regulate the spatial distribution of GFs by controlling the extent of GF binding to the cell matrix [30]. Furthermore, GF degradation in vivo can occur due to denaturation, oxidation or proteolysis [15].

There are many GFs classified in various families based on their target cells, functions, structures and molecular evolution. Jagged/Delta/Serrate/Notch families, the EGF family, the FGF family, NTs, the hedgehog family, the insulin-like growth factor (IGF) family, the hepatocyte growth factor (HGF) family, the hepatoma-derived growth factor (HDGF) family, the connective tissue growth factor (CTGF) family, the platelet-derived growth factor (PDGF) family, the VEGF family, the interleukin-1 (IL-1) family, the IL-6 family, the IL-10/interferon family, the IL-12 family, the IL-17 family, and the Wingless and interleukin-1 (Wnt) families are examples of this [240]. EGF, IGF, and HGF are used in regenerative medicine applications [191]; HDGF is involved in liver development and regeneration [241], whereas CTGF is a potential diagnostic, prognostic and therapeutic biomarker [242], and PDGF can be useful as a potent therapy for heart failure [243]. The IL-2 family is successfully applied in cancer immunotherapy [244].

It is crucial to use GFs in a regulated way to prevent adverse effects, as an excessive production of active GFs can be the reason for cancer. Thus, both GFs and their receptors have become targets for drugs in the redundant growth of cancer cells. The sophisticated engineering of delivery matrices made of biopolymers can provide a dramatic enhancement of GF therapeutic efficiency owing to specific physical properties. The degradation kinetics of polymer-based delivery systems inside the cell matrix enables the monitoring of the control release profile of growth factors, resulting in optimized GF concentrations, which is the main goal of these systems [30].

The design of the GF delivery system is challenging due to its thermal and pH instability, as well as sensitivity to proteolytic degradation [25]. The application of dedicated GF delivery systems is vital for achieving maximum biological efficacy. Such a system should provide spatiotemporal control over GF release and minimize its degradation, while maintaining its bioactivity. GF carriers can be fabricated of synthetic and natural macroions, either alone or in combination [25]. GFs can be encapsulated in a 3D polymer matrix to effectively prolong GFs bioactivity or be attached to MM [34]. It is worth noting that MM containing the GFs are efficient for wound healing [22,245] and tissue engineering [246,247]. Moreover, such a system can act as their natural matrix, with the potential for synergistic therapeutic effects [248]. Thus, drug delivery systems for the effective delivery of various GFs attracted great interest and are the subject of many scientific articles [34,239,249].

#### 2.3.1. NTs

NTs are structurally and functionally related proteins belonging to the cystine knot growth factor family. Pro-NTs are synthesized in vivo and are then cleaved to generate mature NTs by proteases (furin and proconvertase) in a Golgi apparatus or secretory vesicles [250]. The precursors consist of an N-terminal prodomain and a C-terminal mature domain. After translation, the precursors form noncovalent dimers via interactions of the mature domain. The mature NTs are noncovalent homodimers that contain a special three-dimensional structure, known as the cysteine knot. The cysteine knot consists of three disulfide bonds that form a true knot of the polypeptide chain [251]. Mature NTs are released from neurons to the cellular cavities. They play an important role in the development and maintenance of the vertebrate nervous system by promoting the survival, migration, proliferation, differentiation, and death of neurons [252,253]. They are responsible for the regulation of neuronal activity as well as the protection and recovery after neurodegenerative diseases such as stroke and traumatic brain injury [254]. NTs are important in tissue regeneration and repair [255].

NTs such as nerve growth factor (NGF), brain-derived neurotrophic factor (BDNF), neurotrophin-3 (NT3) and neurotrophin-4/5 (NT4/5) were identified in mammals [252]. They exist in the human brain, forming stable, noncovalent dimers with a molecular mass in the range of 13 kDa (NGF) to 27 kDa (BDNF, NT3) and have high IEP (9–10.5) [256]. NTs signal mainly through the tropomyosin-related kinase (Trk) family of tyrosine kinase receptors. NGF signals preferentially through TrkA, BDNF and NT4 through TrkB, and NT-3 through TrkC. NTs regulate the survival of neurons and prevent cell death by combining with Trk. Trk receptors are strongly associated with central and peripheral nervous system processes such as memory, pain, depression, neuronal development, plasticity and protection. TrkA, TrkB and TrkC share significant sequence homology and domain organization [257]. Structurally, Trk proteins contain extracellular and intracellular regions separated by a single transmembrane domain. All three Trk proteins share a high degree of structural homology, including the three leucine-rich motifs and two immunoglobulin-like C2 type domains. TrkB is primarily expressed in the central nervous system, while TrkA and TrkC are both expressed at high levels by the peripheral nervous system [250]. It is worthy of note that TrkA and TrkC are ligand-dependent receptors, whereas TrkB is independent of the NTs concentrations. Thus, TrkB (contrary to TrkA and TrkC) does not induce neuronal apoptosis in the lack of NTs [250].

Furthermore, all NTs interact with equal and low affinity with a member of the tumor necrosis factor receptor (TNFR) superfamily: the p75 receptor [256,258]. This is a high-affinity receptor for pro-NTs rather than mature NTs. p75 is a type I membrane protein (N-terminal outside of the cell) with an extracellular region that is very rich in cysteine residues, and an intracellular region without catalytic activity. p75 plays roles in regulating cell survival, neurodegeneration, and cell death. Furthermore, by activating p75, NTs could induce apoptosis in several cell populations. More information concerning the structure and the biological properties of the p75 receptor can be found in [259].

NGF is a protein with a molecular mass of 13 kDa, consisting of 118 amino acids [255]. It is composed of three subunits, called α, β and γ [260,261]. NGF is produced by the cleavage of its precursor (precursor nerve growth factor, pro-NGF), whose function is different from that of mature NGF [262]. The NGF and its receptors are crucial for the development of the peripheral nervous system and central nervous system as well as the immune system of adult organisms, bone metabolism and regeneration. NGF regulates the embryonic development of peripheral nervous system sensory and sympathetic neurons from the neuronal crest. It was found that phenotypic knockout of NGF in adult mice produces animals with skeletal muscle dystrophy and a reduced number of splenocytes. Moreover, these mice have smaller superior cervical ganglia and a reduced number of dorsal root ganglia neurons compared with wild-type mice [261]. Cell differentiation, survival and proliferation are induced when tyrosine kinase A is activated. If the p75 neurotrophin receptor is bonded to NGF, apoptosis occurs [260]. An IEP of 10.5 was also found for NGF [263].

BDNF was first isolated from the pig brain in 1982 [264]. The precursor protein of BDNF, of a molecular mass of 32–35 kDa, is synthesized in the endoplasmic reticulum. It is then cleaved by a distinct protein convertase enzyme, forming the mature BDNF of a molecular mass of 13 kDa [265]. A biologically active BDNF homodimer has a molecular mass of 27 kDa. It consists of 120 amino acids and forms three disulfide bridges [266]. It is a highly positively charged protein, with an IEP of 10–10.9. The electric charge over BDNF molecules is heterogeneously distributed [266].

Interesting results were obtained from studying the interactions of two synthetic peptides that are able to mimic the proliferation ability of NGF and BDNF with gold surfaces [267]. The peptides strongly interact with each other at pH 7.4, whereas negligible interaction between them was observed in acidic conditions. The proteins, as well as their complexes, were irreversibly adsorbed on the gold substrates. Competitive peptide adsorption was also observed.

NT3 consists of 119 amino acids and possesses an IEP of 9.5 [268]. The kinetics of adsorption of NT3, from low bulk concentration (50 mg/mL), was successfully determined on a modified silica biosensor by Matatagui et al. [269]. NT4/5 molecules form non-covalent homodimers. The molar mass of NT4/5, calculated from the amino acid composition, is 14 kDa. The physicochemical characterization of NT4/5 in bulk and the kinetics of adsorption and desorption on mica were determined by Dąbkowska et al. [253]. The average hydrodynamic diameter of the NT4/5 homodimer was equal to 4.5 nm for a broad range of pHs [253,270]. The protein was positively charged until pH 8.1 (IEP of NT4/5). It was found that NT-4/5 adsorption is governed by electrostatic interactions. The stable NT4/5 monolayer on mica was created at pH 3.5 and 7.4, and for the ionic strength of 0.15 M. The transition between irreversible and reversible regimes was found for the low surface coverage monolayer and the high pH of the rinsing solution [253].

One should notice that despite the obvious advantages, the use of NTs as potential drugs also has limitations related to the age, gender, and therapeutic status of patients, and the presence of the different forms (pro-or mature) of neurotrophic factors [250]. It was found that levels of BDNF, used in major depressive disorder treatment and schizophrenia, are influenced by hormonal status in women [271], whereas in males they were found to be significantly lower than in the control group [272]. Thus, the determination of the correct dose of BDNF for patients is extremely difficult, which limited its application as a drug.

The treatment of neurodegenerative diseases (such as Alzheimer’s disease, Parkinson’s disease, and Huntington’s disease) using NTs also has limitations [273]. NTs are rapidly degraded; thus, they need to be frequently delivered [274]. Moreover, recombinant NT protein cannot pass through the blood-brain barrier [275]; therefore, the drugs have to often be delivered by applying an intracerebroventricular injection [276]. Moreover, significant adverse effects, including anorexia, weight loss, and hyponatremia are sometimes observed after NT treatment [274]. Finally, several failures in the clinical use of neurotrophic factors were reported, where no improvement in ratings of motor signs was observed [274]. The applications of the neurotrophin family in medicine are summarized in Table 4.

#### 2.3.2. FGFs

FGFs are a family of proteins involving 22 members (FGF1-FGF 23), named by their ability to stimulate fibroblast proliferation [15,283]. FGF15 was not identified in humans [283]. By considering the mechanisms of the action, these proteins can be classified as intracellular FGFs, canonical FGFs, and hormone-like FGFs [283] or as intracrine, paracrine and endocrine FGFs [283]. Based on the possible evolutionary relationships, FGFs are also classified into seven subfamilies [283]. The molecular masses of the FGFs are in the range of 17 to 34 kDa, and they share 13–71% amino acid identity [15,283]. Despite the considerable practical significance of FGFs, their structure and physicochemical properties were obtained solely for some proteins.

FGFs are expressed in nearly all tissues, and they play an important role in the earliest stages of embryonic development, as well as in tissue maintenance, repair, regeneration, and metabolism in adults. The FGF family is involved in regulating the biological responses of cell adhesion, angiogenesis, cellular migration, tissue differentiation, the regeneration of damaged tissue of the skin, the formation of the blood vessels, muscles, adiposes, cartilages, bones, teeth, nerves, in wound healing and the metabolism of lipids, sugars and fats [15]. Similar to other GFs, free-FGFs are easily degradable in vivo. This leads to the loss of biological activity and functions [15].

FGFs transmit signals intracellularly through the binding and activation of four signaling tyrosine kinase fibroblast growth factor receptors (FGFRs) [284]. Each receptor has a unique affinity for FGFs. Ligand (FGF)-receptor interactions are modulated by the cofactors heparin and Klotho. When FGFs bind to FGFRs, four key signaling pathways, including mitogen-activated protein kinase (MAPK), the phosphoinositide 3 kinase/AKT (PI3K-AKT), signal transducer and activator of transcription (STAT), and the phospholipase C gamma (PLCγ) are activated to influence gene transcription [284]. These signaling pathways regulate cell proliferation, differentiation, and survival, as well as cellular migration and adhesion dynamics [284]. FGFs play roles in tumorigenesis and pulmonary fibrosis, and have unique capacities to protect against DNA damage induced by oxidants and some environmental toxicants.

It should be noted that FGF signaling, as well as a suitable concentration of FGF serum, are crucial for human life and well-being. Any FGF signaling causes human diseases or metabolism disorders. For example, the mutations of FGF20 can lead to Parkinson’s disease, whereas FGF23 mutation can lead to familial tumoral calcinosis. The increase in the concentrations of FGF21 serum causes type 2 diabetes and obesity [283], whereas a decrease in FGF21 serum concentration occurs in anorexia nervosa [285]. A high concentration of FGF23 serum is responsible for renal failure [283]. FGF mutations are connected with various diseases, including different cancers [286,287].

On the other hand, the suitable concentration of the FGFs in the human body allows them to regulate a broad spectrum of biological functions. Thus, they can be applied in medicine as effective drugs in gene therapy. However, one should be aware that the applications of FGFs for human treatment are not trivial, and can also cause side effects. Unger et al. evaluated the safety and tolerability of FGF2 administered to patients with stable angina pectoris secondary to coronary artery disease [288]. FGF2 caused acute hypotension (10%) that was independent of dose. A total of 20% of patients receiving the high dose of FGF2 had sustained hypotension, whereas 30% developed bradycardia. FGFs promote wound healing; however, individual factors, including sex, may affect the results. When growth factors are applied to necrotic tissue, competitive inhibition at the early stage of wound healing occurs, countering the curative effect of the growth factors [289]. It also found potential deleterious effects of FGF21 on bone homeostasis in rodent models. It can also have an impact on developing human therapies that rely on FGF21 [290]. The examples of the potential applications of the individual FGSs in medicine are summarized in Table 5.

#### 2.3.3. VEGF

VEGF plays an important role in angiogenesis, endothelial cell growth, and proliferation, hypotension, and vascular permeability [326]. In mammals, the VEGF family involves five members: VEGF-A, VEGF-B, VEGF-C, VEGF-D, and PIGF (placental growth factor). Before the discovery of the latter members, VEGF-A was known as VEGF. VEGF-A, also called VPF (vascular permeability factor), was discovered by Folkman et al., who reported a factor secreted by tumours causing angiogenesis and named it tumour angiogenesis factor [327]. This protein is a positively charged homodimer glycoprotein with a molecular mass of 45 kDa that is heparin-binding [326]. It has an IEP of 8.6 and a low clearance half-life of less than 1 h following injection in vivo [34].

The VEGF family contributes to neoangiogenesis, vasculogenesis, apoptosis inhibition, vasodilation, cell proliferation, and vascular permeability. It is effective in revascularization and tissue recovery; thus, it can be used in therapeutic angiogenesis aiming to deliver it to ischemic or injured tissues to promote the targeted formation of new blood vessels [326]. VEGF is essential for physiologic vascular homeostasis in diverse cells and tissues. It is important in the molecular pathogenesis of tumour growth and metastasis, as well as in retinopathy connected with several blinding eye diseases [328]. Those pathogenic effects are primarily due to VEGF effects on vascular permeability and neoangiogenesis. The incorrect concentration of VEGF in the serum is responsible for several diseases, e.g., Alzheimer’s disease [329], cardiovascular diseases [330,331], coronary heart disease [332], Lyme disease [333]; kidney diseases [334], and eye diseases [335].

## 3. Macroion Layers and Macroion Complexes in Growth Factor Delivery

Macroion assemblies form an efficient scaffold for GF adsorption. Such assemblies enable the targeted delivery of these proteins without losing their activity.

### 3.1. PAH-Based Assemblies

PAH-based assemblies were applied for rapid tissue integration and to avoid prosthetic rejection stimulation of transprosthetic vascularization. For this purpose, the surface of porous titanium implants was modified by PAH/PSS (poly(sodium-4-styrenesulfonate) films functionalized with VEGF [336]. The VEGF adsorbed on the (PAH/PSS)_4_ multilayers maintained its bioactivity in vitro and stimulated endothelial cell proliferation. NTs such as BDNF incorporated into the PAH/PSS films remained functional forming functionalized nanofilms [337].

### 3.2. BPEI-Based Assemblies

BPEI was successfully applied as a carrier for delivering VEGF isoforms. An increasing factor transfection efficiency without a lowering of cell viability was observed [338]. Using bPEI as a carrier revealed the possible application of VEGF in gene therapy for the treatment of wounds and cardiovascular diseases. The same complex type can be applied as a potential agent for the treatment of myocardial ischemia. The efficacy of that complex was compared with PAMAM-VEGF. However, the bPEI complex induced lower relative viabilities of cells by half compared to that formed by PAMAM-VEGF (95%) [339].

Interesting results based on the coacervation of VEGF with PEI and CS were reported by Huang et al. [340]. VEGF encapsulation efficiency (∼85%) and GF release (10 days) depended on polycation type and were the highest for the complexes based on CS. However, both VEGF-based coacervates effectively stimulated endothelial cell proliferation.

### 3.3. PAMAM Dendrimer-Based Assemblies

PAMAM dendrimer-covered surfaces have shown to be a suitable platform for the grafting of VEGF, FGFs and NTs [58,126,128,266,270,339,341,342,343]. VEGF-PAMAM complexes were successfully applied in theranostics for inducing the apoptosis of cancer cells as well as the inhibition of tumour cell growth [342,343]. It was found that the bioconjugates were effective in cardiovascular disease treatment [339] and wound treatment [128]. Furthermore, the VEGF-PAMAM assemblies were successfully used in the boron neutron capture therapy (BNCT) of cancer, as was presented in the pioneering work of Backer et al. [343]. The authors found that fluorescently labelled PAMAM dendrimers, equipped with 102 to 110 decaboranes and VEGF, accumulate in the tumour periphery in vitro where angiogenesis was most active. Thus, these bioconjugates can be effective as a targeting agent for the BNCT of the tumour neovasculature. Arginine (ARG)-grafted PAMAM dendrimers combined with plasmid DNA encoding VEGF led to the development of an effective method to treat diabetic skin wounds, as demonstrated by Kwon et al. [128].

Besides the PAMAM-VEGF coacervates, the complexes based on the FGFs can be applied for wound healing, as reported by Thomas et al. [126]. It was also shown that the PAMAM-FGF1 complexes can serve as a platform for cytosolic and nuclear drug delivery in tumour cells, and as an FGF delivery agent for angiogenesis. The conjugation of PAMAM dendrimers with peptides obtained from FGF3 allowed for the formation of a novel PAMAM-based vector with enhanced gene expression efficiency [341]. The heparin-PAMAM assemblies effectively bind FGF2, which enables the design of new anti-inflammatory drugs with minimal side effects [58].

Dąbkowska et al. applied complexes based on PAMAM dendrimers for the continuous delivery of the NTs for the treatment of neurodegenerative disorder [266,270]. The detailed physicochemical characteristics of the assemblies were investigated, and the sizes, zeta potentials, and the stabilities of obtained complexes were determined. They were formed by generation 5.5 PAMAM and BDNF [266], as well as generation 6 PAMAM and NT 4/5 [270], respectively. Both were negatively charged in physiological conditions. The obtained results show great potential for the design of stable drug-delivery systems that are crucial for the neuroprotection and treatment of damaged retinal neurons.

### 3.4. PAE and PAA-Based Assemblies

MM based on PAE and polyanion PAA allow for the composing of biocompatible and functional nanofilms with high loading efficiency and short buildup times. Such MM were also successfully applied for the loading and releasing of active FGF2 [344]. In the follow-up studies, the P.T. Hammond group constructed LbL films with tunable VEGF delivery via degradable PAEs and PAA. It resulted in no burst release of the GF. The release of VEGF was discharged from the MM after just 8 days [249].

### 3.5. CS-Based Assemblies

**Figure 4:**
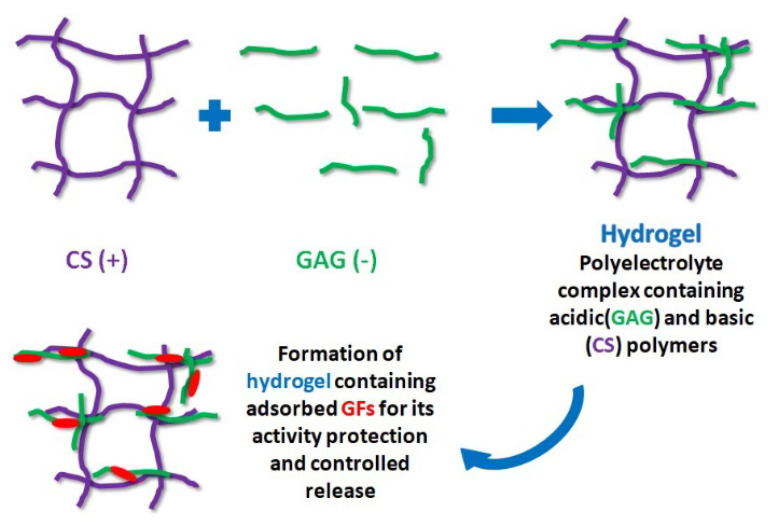
Polyelectrolyte complex containing CS (violet chains) and GAG (green chains) as a substrate for adsorption, activity preservation, and the controlled release of GFs (red macromolecules).

CS-based scaffolds were examined in terms of GF incorporation and delivery [345,346]: heparin functionalized CS–alginate scaffolds with FGF2 for tissue regeneration [347], CS/collagen composite scaffold containing recombinant human bone morphogenetic protein-2 (rhBMP-2) for dental implant osseointegration [348], a brushite–CS system, which controls the release kinetics of incorporated VEGF to enhance bone healing [349] as well as in the form of macroion complexes (hydrogel) with negatively charged GAGs [350]. The formation of hydrogel with GAGs is schematically presented in Figure 4.

CS was also evaluated as local implants in the form of microspheres and fibres for sustained release depots of endothelial growth factors [346].

### 3.6. Heparin-Based Assemblies

Heparin is one of the most commonly used macroions for FGF binding. Binding heparin to FGF2 improves the protein bioactivity and stability, highly increases GF mitogenic potential, and preserves FGF2 from heat, pH changes, and proteolysis [13]. Heparin/ChS films also enhanced FGF2 activity. FGF2 released from these MM retained their in vitro activity and promoted the proliferation of preosteoblast cells [13]. When FGF1 is incorporated into the heparin/bPEI multilayers, it enhances the viability and proliferation of the fibroblasts [351].

The effective adsorption of FGF2 on heparin-terminated MM is reported in Refs. [352,353], where the adsorption kinetics and the maximum surface concentration (120 ng/cm^2^) of the GF were determined in physiological conditions. Furthermore, the FGF2 layer was found to be bioactive, and stimulated both the proliferation and the differentiation of the calf pulmonary arterial endothelial cells [353]. In addition, a greater cell density and a higher proliferation rate of mesenchymal stem cells than any of the other tested conditions were observed when FGF2 was adsorbed onto heparin-terminated multilayers [354]. The biological activities of the FGF2 and heparin released from decellularized porcine aortic heart valve leaflets were confirmed by De Cock et al. [355]. The release of heparin and FGF2 from the scaffold under physiological conditions was sustained over 4 days while preserving the biological activity of the released GF.

The heparin-based MM were also applied for the controlled immobilisation of NTs: NGF and BDNF. Such platforms show enhanced neurite outgrowth in comparison to control surfaces [356]. VEGF is also effectively adsorbed on the top of MM terminated by heparin. When the MM is formed by CS/alginate/carrageenan/heparin, VEGF creates an active layer for human adipose-derived stem cell proliferation [215]. The coacervates based on heparin-binding peptides were applied for providing the prolonged release of NGF, BDNF and NT3 to nerve regeneration by Sakiyama-Elbert [357].

### 3.7. ChS-Based Assemblies

The effective adsorption of FGF2 on MM, with ChS as the outer layer, was successfully developed by Tezcaner et al. [358]. The researchers showed that FGF2 deposits on ChS-terminated films can stimulate the attachment of photoreceptor cells and maintain the differentiation of rod and cone cells. The wettability of ChS/FGF2 multilayers was also determined [12]. The measurements revealed that the ChS layer (with a contact angle 55°–60°) is more hydrophilic than the FGF2 layer (with a contact angle 65°–70°). Moreover, the FGF2 layer adsorbed on ChS was stable. Only 30% of the incorporated FGF2 was released within 8 days. Surprisingly, it was found that collagen layer covered ChS/FGF2 films have a better ability to stimulate fibroblast proliferation than FGF2, where it served as an outer layer.

Interesting results relating to the interactions of FGF1 and FGF2 with heparin, ChS and λ-carrageenan were obtained by Sun et al. [59]. Those polysaccharides effectively attached FGF1 and FGF2 and increased their thermal stability for a longer time. Thus, it was confirmed that the polysaccharides bind and stabilize FGFs, and also potentiate their activity and control their delivery.

### 3.8. Hydrogel-Based Polysaccharides Containing GFs

Hydrogel formation is a novel strategy in wound dressing, since loading growth factors into a hydrogel to construct a sustained-release system is considered a promising approach to improve wound healing [359]. Such a material not only promotes tissue regeneration but also prevents skin scarring. Hydrogel-based polysaccharides also provide the stability of GFs and their effective delivery. For example, CS, heparin and HA-based hydrogels form assemblies with VEGF, FGFs and NTs [357,360,361,362], where the activity of GFs [360,361] is enhanced.

FGF1, FGF2 and VEGF have been incorporated into CS hydrogels, where they induced neovascularization [360] as well as contraction and acceleration of wound closure [361]. Both FGFs and VEGF, trapped in the hydrogel, remained in their active form and accelerated the proliferation of the cells [360]. Hydrogels based on heparin allow for controlled VEGF release and promote the healing of diabetic wounds [362].

ChS, heparin, and HA were applied for the formation of biocompatible hydrogels for wound repair [363]. Those biomaterials controlled the release FGF2 in vivo. It was found that the released amount of FGF2 increases with lower percentages of heparin. In addition, efficient neovascularization was determined for the hydrogel containing ChS, heparin, HA and FGF2. Furthermore, it was found that the release rate of FGF2 from MM increases with decreasing pH [364].

Jha et al. [365] developed a series of hydrogels based on heparin-functionalized HA and investigated the effect of heparin molecular weight as well as its relative concentration on the loading efficiency and retention behaviour of the GF. The results demonstrated that gels based on heparin with high molecular mass facilitated GF loading and retention. Moreover, HA-based hydrogels functionalized with HMW heparin effectively bind GF and induce a more robust differentiation of stem cells into endothelial cells, which further stimulated the vascular-like network formation within the hydrogels.

The coacervates based on HA were successfully applied as VEGF carriers [366,367]. Parajó et al. used HA/CS gel nanoparticles for the delivery of VEGF [366]. The obtained HA/CS system, exhibiting good stability and low cytotoxicity, was able to entrap (association value of 94%) and release VEGF within 24 h. VEGF was also attached to nanogels containing HA to induce angiogenesis in order to prevent heart failure [367]. The authors found that the system was biocompatible and significantly improved angiogenesis. HA-heparin conjugate gel, formed by amine-modified HA bound to oxidized heparin, was also applied for the binding and controlled release of FGF2 [368]. It is worth noting that released FGF2 was biologically active in stimulating cell growth in vitro. Besides FGFs, HA-based gels were applied for the efficient delivery of NTs, such as BDNF, that demonstrate the utility of HA-based coacervates as a platform for localized gene therapies after spinal cord injury [369]. The macroion/GF assemblies for biomedical applications are summarized in Table 6.

## 4. Conclusions

GFs are a naturally occurring heterogeneous group of proteins or polypeptides capable of stimulating cell proliferation and differentiation, as well as potentially being used in treatments for neurodegenerative diseases and in wound healing. These molecules play critical roles in both normal and abnormal processes within a living organism. However, due to their short effective half-life, low stability, and susceptibility to enzymatic degradation at typical body temperatures, they are rapidly degraded in vivo. To overcome these issues, macroions have been suggested as carriers to deliver and control the release of GFs. Biocompatible macroions have been demonstrated to be effective scaffolds for binding and stabilizing growth factors, as well as controlling their release and avoiding potential side-effects. In addition, these macroions can imitate the ECM environment to provide a supportive environment for GFs. Moreover, macroion films derived from polysaccharides possess remarkable blood compatibility. Hydrogels of polysaccharide origin have been shown to support cell growth and proliferation. Furthermore, a number of drug delivery systems based on polysaccharides have been designed to effectively deliver GFs, increasing their stability and providing sustained release.

On the other hand, it is crucial to be aware of the limitations in the use of biocompatible macroions. They mostly belong to pH-responsive macromolecules; thus, they can act within the specified (mostly 4–8) pH range. Therefore, their role in the efficient delivery of drugs (under pH-dependent body conditions) into a stomach (pH 1.5 to 2.0) and a colon (pH 7.9 to 8.5) is limited. Most of the synthetic macroions are stable within the low ionic strengths (up to 0.15 M), whereas they form aggregates in higher ionic strengths. Moreover, the cytotoxicity of dendrimers depends on the type of groups at the rim. Half-generation PAMAM dendrimers, possessing carboxylate groups, are much less cytotoxic than the dendrimers with only NH_2_ groups. Many of the therapies based on complexes are expected to have large market sizes and to require high concentrations of macroions, with precisely defined molar masses and low polydispersity.

In order to be effective, macroions must feature certain biological and physicochemical properties, such as biocompatibility, a high affinity for binding GFs, improved bioactivity and stability of the GFs, protection from heat, pH changes, proteolysis, and the appropriate electric charge for GF attachment via electrostatic interactions, among others. It is further essential for macroions to avoid aggregation in different types of environments. Finally, the macroion-based carriers should allow GFs to sustain an effective and lengthy lifetimes and to release the cargo in a controlled manner.

In this review, natural (only polysaccharides) and synthetic macroions were discussed, and their basic physicochemical properties and applications were explored. The parameters such as typical conformations, IEPs, zeta potentials, and sizes in different environmental conditions were also examined. Moreover, the advantages and disadvantages of such macroions were discussed. Additionally, the use of macroions, particularly as GF carriers, was emphasized. It is worth noting that the scientific literature surrounding the physicochemical properties of GFs is limited.

Three major groups of growth factors (VEGF, FGFs, and NTs) were discussed in detail, outlining the basic physicochemical properties and medicinal applications. The formation process and the physicochemical and biological properties of biocompatible macroion/growth factor assemblies were reviewed, while their applications in the medical field were highlighted.

Going forward, the focus of research should be the determination of the physicochemical properties of the growth factors as a function of temperature, ionic strength, pH, and the addition of simple ions of various valences. In terms of theoretical investigations, future studies should be dedicated to elucidating the role of the electric double layer, specific ion adsorption, and local pH changes on the properties of GFs immobilized on various substrates. Additionally, further investigations should be conducted to clarify the mechanisms of the binding of GFs with potential biocarriers, as well as to develop more effective methods for delivering these proteins, which are of great importance for the diagnosis and treatment of neurodegenerative diseases and chronic wound healing.

**Figure 5:**
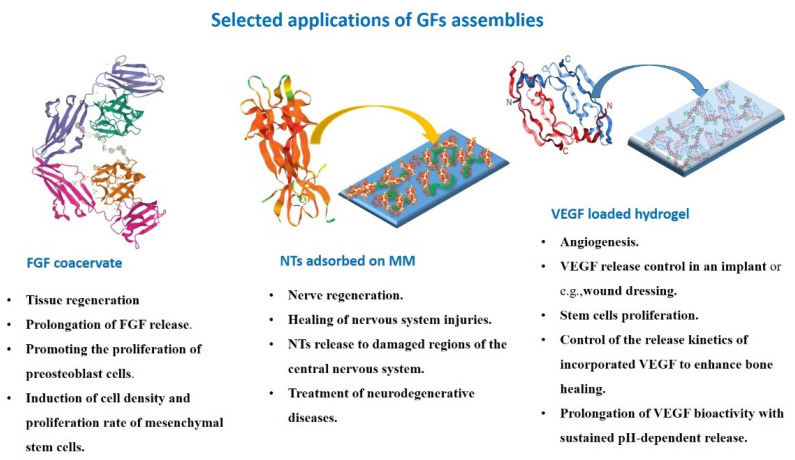
Various macroion/growth factor assemblies and their applications.

For the convenience of the reader, the main applications of GFs are collected and presented in Figure 5.

## 5. Perspective

There are still many questions that remain to be answered in the future. An example of this is the designing of low-cost, monodisperse synthetic macroions that are stable in a broad pH range and high ionic strength, and that are of high biodegradability and cytocompatibility compared with polysaccharides. On the other hand, these new synthetic, biocompatible macroions, unlike polysaccharides, should be highly charged and soluble in aqueous solutions in a broad pH range. These macroions will allow for the more effective binding and releasing of GFs. Furthermore, using the macroion-based materials requires greater emphasis on developing and testing macroion cell modification in clinically-meaningful models, and ultimately in humans. The investigation of the mechanisms of synthetic macroions’ cytotoxicity is crucial to identify critical parameters in order to design promising materials in future biomedical materials.

The biocompatible macroion/growth factor assembly undoubtedly has the potential to be used in medicine, and not only for wound healing or tissue regeneration. NTs and FGFs can be applied as effective ocular drugs to treat ophthalmic disease, in the treatment of mental (post-traumatic stress disorder or depression) disorders, other conditions (obesity, rickets and osteoporosis), or neurodegenerative diseases.

## Figures and Tables

**Table 1:** The advantages and limitations of macroions for biomedical applications.

	Synthetic Macroions	Polysaccharides
Advantages	Defined size, shape, charge density, structure, mechanical properties [43,44,45], High purity, quality, reproducibility, high mechanical stability [4,45] Biocompatibility [45] Low polydispersity index [43] High solubility in water [46] Well-solubility in solvents of various polarity [47] Broad working range of pH and ionic strength [4,48,49,50] Low production cost [4,45] Non-biodegradable and toxic [4,45] Complicated synthesis, high production cost [45] Limited bioactivity [4] Possible harmful degradation products [4,45]	Natural macroions (biomimetism) [4,9,51] Easy accessibility, easy processing [9,45,51] Excellent biocompatibility, biodegradability, low toxicity, non-immunogenicity [4,9,51,52] Interactions with biomolecules [4,13,21,22] Hydrogel formation [4] Broad or mixed molecular mass [51,52] Low charge density and poor solubility in most organic solvents [4,51,52] High polydispersity index [4], Limited working range of pH and ionic strength [4,53] Various solubilities in water [54] Poor batch-to-batch reproducibility, degradation tendency, potential antigenicity [45] Complicated extraction, high production cost [45]
Limitations		

**Table 2:** Focus and major findings.

Focus and Major Findings
Review on physicochemical properties of polysaccharide-based films [4] Effective functionalization and construction of carbohydrate nanocarriers for biomedical applications [9] Enhancement of FGF2 activity by incorporation of heparin and ChS into the macroion film [12] Enhancement of the proliferation and migration of skin-related cells induced by administration of GFs via coacervates [21] Effective chronic wound treatment by using the novel delivery system based on gelatin/sodium alginate/epidermal growth factor (EGF) assemblies [22] Review on molecular-level control of size, shape, surface chemistry, topology, and flexibility of starburst dendrimers [43] Review on physicochemical properties of branched and linear poly(ethylene imine)-based conjugates and their applications in gene delivery [44] A book on the role of macroions in the drug delivery system [45] Review on the role of polysaccharides [51] and synthetic polypeptides [55] in functional drug delivery Review on strategies to modify polysaccharides for the development of polysaccharide-based biomaterials [52] Review on kinetics and mechanism of PDADMAC and its copolymers syntheses, chemical structures, behavior in solution, molecular characterization, and interactions in solution and at interfaces [46] Solvent quality for the hydrophobic macroions in aqueous solutions can be improved by adding to water a miscible organic good solvent [47] Streaming potential measurements are useful for determining adsorption mechanisms and physicochemical characteristics of MM on solid surfaces, especially their stability [48] PDADMAC and poly-L-lysine (PLL) monolayers are more stable than PAH layer [49] PAMAM dendrimer layers can be exploited for studies of their acid-base properties [50] LBL technique can be used to tune the structure of heparin/CS films [53] Polysaccharides are more soluble than purely homogeneous macroions. Ionized linear polysaccharides easily form gels [54]

**Table 3:** Physicochemical and biological properties of the biocompatible macroions.

	Macroion	Physicochemical Properties	Biological Properties
Synthetic macroions	PDADMAC	strongly positively charged hydrophilic macrocation [70]	water treatment [46]
		effective ionization degree: 13–8% [70]	dental material component [69]
		linear shape [70]	DP gelator formation [65]
		“side-on” adsorption (low ionic strength) [70]	‘‘anchor layer’’ formation [66]
		formation of loops and tails (high ionic strength) [73]	
	PAH	weak polybase, high-pH-dependent charge, prolate spheroid (moderate ionic strength), semicircle (high ionic strength) [75]	growth factors carriers [80]
		“side-on” adsorption [75]	bioimaging, drug carrier [18,81,82]
		reversible swelling/shirking of layer [76]	enhancement of adhesion of proteins and cells [76]
	PAH derivatives	strongly charged macroions [83]	gene delivery carriers [83]
			controlled drug release [84]
			photoreactive growth factor formation [85]
	PAEs	Thermoresponsive [87]	gene/drug carrier [89,90]
		Polydisperse [86]	tissue engineering [86]
	bPEI	weak polybase with primary, secondary and tertiary amino groups [98]	plasmid DNA vector [103]
		spherical shape in solution, flatten after adsorption [101]	growth factor carrier [104]
	PAMAM dendrimers	monodisperse, nano-sized, radially symmetric, pH-dependent charge [106]	genes/drugs nanocarriers [106,109,110]
		easy modification of terminal functional groups [107]	lung diseases treatment [110]
		molecules flatten after adsorption [50,121]	amyloidogenesis disturbing [127]
		pH dependent conformation changes [122]	treatment skin wounds and infection treatment [128,129]
		swelling/shrinking behavior [115,118,119,123]	drug solubility enhancement [137]
	PAA	weak, linear polyacid [144]	growth factor [155]
		flexible rod (high ionization degree) and sphere (low ionization degree) formation [145]	drug carriers [152]
		“side-on” adsorption (low molecular mass); formation of loops and tails (higher molecular mass) [147]	
		ionic strength and molecular mass layer thickness dependency [150,151]	
Natural macroions (polysaccharides)	CS	positively charged, well soluble in an acidic medium; poorly charged, insoluble at high pH [165]	drugs/growth factors/stem cells/peptides carriers [160]
		formation of rods, random and stiff coils in bulk [168]	surface-induced thrombosis and blood coagulation enhancement [173]
		“side-on” adsorption, formation rigid and thin layers (low pH) and thick layers and gels (neutral pH) [169,170]	tissue growth [174]
			wound healing supporting [185]
	HA	highly hydrophilic polyanion, loose hydrated network formation [236]	drug/gene carriers [237]
		stiff coil in aqueous solution [238]	cytokines/chemokines/growth factors production stimulation [25]
			drug carriers [184]
			anticancer therapy [183]
	Heparin	highly negatively charged, high polydispersity [186]	involved in cell adhesion, migration, proliferation differentiation, effective anticoagulant and anti-inflammatory agent [187]
		elongated shape [186,195]	lipid transport, clearance, wound healing [186]
		acceleration or inhibition of protein adsorption depending on protein charge [76,190]	FGFs and VEGF binding [186]
			angiogenesis inhibitor [189]
			FGF carriers [57]
			cell adhesion increasing [76]
	λ-carrageenan	high molar mass [204,205]	drug delivery and release [196]
		negatively charged, high solubility in water [219]	inhibitors of viruses and bacterial infections [158,197,198,199]
		extended and flexible conformation in bulk [204,205]	growth factor binding inhibitor [208]
		“side-on” adsorption (low initial bulk concentrations), highly hydrated quasi “polymeric brushes (high bulk concentrations) [219]	HPV acquisition prevention, HIV inhibitor [158,197]
			protection of growth factors against denaturation [59]
			antibacterial properties [199]
	ChS	linear shape, negatively charged [222,223]	bone healing and growth, osteoarthritis treatment [220,225]
			growth factor binding [222,226]

**Table 4:** The application of the neurotrophin family in medicine.

Neurotrophin Type	Application in Medicine	Ref.
NGF	attenuation of chronic pain behaviour in osteoarthritis, relieving symptoms and promoting healing in ophthalmological diseases (neurotrophic keratitis and dry eye disease)	[277,278]
BDNF	recovery from brain injury, treating deafness, potential drug for Parkinson’s disease treatment	[279,280,281]
NT3	nerve regeneration, spinal cord injury treatment, potential hypoglycemic agent	[254,265,282]
NT4/5	retinopathies treatment	[252]

**Table 5:** The potential application of FGFs in medicine.

FGF Type	Application in Medicine	Ref.
FGF1	treating neuropathic pain	[291]
	wound repair	[292]
	improving cardiac function (new blood vessels in the damaged heart)	[293]
	diabetic retinopathy treatment	[294]
FGF2	wound repair	[292]
	surface modification and restoration of bone defects	[295]
	tissue engineering	[246]
	dermal filler	[296]
	regenerative endodontic procedures	[175]
	muscle regeneration	[297]
FGF3 (expressed in embryonic development)	regulation of the guidance of thalamocortical axons	[298]
FGF4 (expressed in embryonic development)	angiogenic gene therapy	[299]
FGF5	potential therapeutic medication for alopecia (based on FGF5 mutant protein)	[300]
FGF6	muscle development and regeneration	[301]
	preventing and treating metabolic diseases	[302]
FGF7	signal mediator during liver regeneration	[303]
	wound healing acceleration	[304]
FGF8 (expressed in embryonic development)	promote healing of the dental pulp	[305]
FGF9	Parkinson’s and Huntington's disease therapy	[306]
FGF10	wound healing and tissue repair, regenerative medicine (FGF10 enables the differentiation of ES cells)	[287,307]
FGF11	not established	-
FGF12	potential drug in pulmonary arterial hypertension	[308]
FGF13	prognostic biomarker for cancer therapy	[309]
	inflammatory pain treatment	[310]
FGF14	potential drug for the regulation of motor coordination and balance	[311]
FGF15 (expressed in embryonic development)	not identified in humans	[283]
FGF16	protection of the neonatal heart	[312]
	protection of the heart from cancer drug-induced heart dysfunction	[313]
FGF17 (expressed in embryonic development)	potential drug for neuropsychiatric disorder treatment	[314]
FGF18	biomarker for detection of ovarian cancer	[315]
FGF19 (expressed in embryonic development)	potential biomarker for detection of cancer (hepatocellular carcinoma)	[316]
	treatment of muscle wasting	[317]
FGF20	FGF20-based adjuvant therapy to cure Parkinson’s disease	[318]
FGF21	potential biomarker for the early detection of cardiometabolic diseases	[319]
	obesity treatment	[320]
	promoting the recovery after spinal cord injury	[321]
	potential drug for prophylaxis and treatment of thrombotic disease	[322]
FGF22	relieving symptoms of depression	[323]
FGF23	early biomarker of chronic kidney disease	[324,325]

**Table 6:** The applications of the different biomaterials with attached/incorporated growth factors.

Biomaterial	Growth Factor	Application of Biomaterial/GF Assemblies	Ref.
PAH/PSS-based films	VEGF	stimulation of endothelial cells proliferation (pro-angiogenic coating)	[336]
PAH/PSS-based films	BDNF	stimulation of motoneurons adhering and spreading	[337]
bPEI	VEGF	enhancement of VEGF transfection efficiency	[338]
bPEI, PAMAM dendrimers	VEGF	myocardial ischemia and infarction treatment	[339]
PEI and CS- based complexes	VEGF	generation of new blood vessels	[340]
PAMAM dendrimers	FGF1	wound healing	[126]
PAMAM dendrimers	FGF3	enhancement of gene expression efficiency	[341]
PAMAM dendrimers	VEGF	breast cancer treatment	[342]
PAMAM dendrimers	VEGF	anti-cancer therapy	[343]
PAMAM dendrimers	NT4/5	treatment of damaged retinal neurons	[270]
ARG-grafted PAMAM dendrimers	VEGF	treatment of diabetic skin wounds	[128]
PEG-grafted PAMAM dendrimers	BDNF	delivery of neuroprotective proteins	[266]
heparin,carrageenan, ChS	FGF1, FGF2	FGF-based drug delivery	[59]
heparin glycodendrimers	FGF2	tissue repair and regeneration	[58]
heparin-based hydrogel	VEGF	diabetic wound treatment	[362]
heparin layers	NGF, BDNF	formation of bioactive surfaces stimulated neurite outgrowth	[356]
heparin-based films	FGF2	stimulation of aortic heart valve regeneration	[355]
PAE/collagen/heparin/PAA- based films	FGF2	novel platform for the culture of human pluripotent stem cells	[344]
heparin-functionalized CS/alginate gels	FGF2	tissue regeneration	[347]
heparin/HA/ChS-based hydrogels	FGF2	wound repair	[363]
heparin/HA- based hydrogels	FGF2	damaged tissue reparing	[368]
PEI (PSS)/heparin-based films	FGF2	enhancement of bone formation	[13]
bPEI/heparin- based films	FGF1	formation of bioactive materials	[351]
albumin/heparin- based films	FGF2	formation of biofunctional surface coating	[353]
fibrin/heparin-based films	NGF, NT-3, BDNF	enhancement of peripheral nerve regeneration	[357]
CS-based hydrogels	FGF1, FGF2, VEGF	wound occlusive dressings for dermal and peptic ulcera	[360]
CS-based hydrogel	FGF2	wound dressing	[361]
CS/heparin- based films	FGF2	formation of biofunctional surface coating	[354]
quaternized CS/heparin-based films	FGF2	formation of biofunctional surface coating	[352]
CS/alginate/carrageenan/heparin	FGF2, VEGF, PDGF	tunable incorporation of platelet lysate	[215]
CS/HA nanoparticles	VEGF	treatment of ischemia-related diseases	[366]
CS/ collagen-based films	rhBMP-2	enhancement of implant osseointegration	[348]
brushite/CS-based films	VEGF	bone regeneration	[349]
PAE/ChS/PAA-based films	VEGF	bone tissue engineering	[249]
PLL/ChS and PLL/HA- based films	FGF2	functionalized tissue engineering	[358]
ChS/collagen-based films	FGF2	formation of biofunctional surface coating	[12]
HA- based hydrogels	VEGF	drug delivery system	[367]
HA- based hydrogels	BDNF	localized gene therapies	[369]

## Data Availability

Not applicable.

## Abbreviations

Abbreviations	Definition
AFM	atomic force microscopy
ARG	arginine
ASOs	antisense oligonucleotides
BDNF	brain-derived neurotrophic factor
BMP-2	bone morphogenetic protein-2
BNCT	boron neutron capture therapy
bPEI	branched polyethyleneimine
CC	complex coacervates
CG	coarse-grained
ChS	chondroitin sulfate
CS	chitosan
CTGF	connective tissue growth factor
DA	acetylation degree
DP	dendronized polymer
ECM	extracellular matrix
EGF	epidermal growth factor
FA	folic acid
FGF	fibroblast growth factor
FGFR	fibroblast growth factor receptors
FR	folate receptor
GAGs	glycosaminoglycans
GF	growth factor
HA	hyaluronic acid
HDGF	hepatoma-derived growth factor
HGF	hepatocyte growth factor
HIV	human immunodeficiency virus
HMW	high molecular weight
hNPCs	human neural progenitor cells
HPAEs	hyperbranched poly(β-amino ester)
HPV	human papillomavirus
HSPGs	heparan sulphate proteoglycans
IEP	isoelectric point
IGF	insulin-like growth factor
IL-1	interleukin-1
IL-6	interleukin-6
IL-10	interleukin-10
IL-12	interleukin-12
IL-17	interleukin-17
LbL	layer-by-layer
LMW	low molecular weight
LMWH	low molecular mass heparin
lPEI	linear polyethyleneimine
MALS	multi-angle light scattering
MAPK	mitogen-activated protein kinase
MD	molecular dynamics
MM	macroion multilayers
NGF	nerve growth factor
NT	neurotrophin
NT3	neurotrophin-3
NT4/5	neurotrophin–4/5
PAA	poly(acrylic acid)
PAEs; PBAEs	poly(β-aminoesters)
PAH	poly(allylamine hydrochloride)
PAMAM dendrimers	polyamidoamine dendrimers
PAMAM-COO	PAMAM dendrimers with carboxylic groups
PAMAM-NH_2_	PAMAM dendrimers with primary amines
PDADMAC	poly(diallyldimethylammonium chloride)
PDGF	platelet-derived growth factor
PEG	polyethylene glycol
PI3K-AKT	phosphoinositide 3 kinase/AKT
PIGF	placental growth factor
pK_a_	negative base-10 logarithm of the acid dissociation constant
PLCγ	phospholipase C gamma
PLL	poly(L-lysine)
pro-NGF	precursor nerve growth factor
PSS	poly(sodium-4-styrenesulfonate)
QCM	quartz crystal microbalance
RES	reticuloendothelial system
rhBMP-2	recombinant human bone morphogenetic protein-2
SANS	small-angle neutron scattering
SARS-CoV-2	severe acute respiratory syndrome coronavirus 2
SAXS	small-angle X-ray scattering method
STAT	signal transducer and activator of transcription
TNFR	tumor necrosis factor receptor
Trk	tropomyosin-related kinase
VEGF	vascular endothelial growth factor
VEGF-A; VPF	vascular permeability factor
Wnt	Wingless and interleukin-1
XPS	X-ray photoelectron spectroscopy
